# Integrated meta-analysis of colorectal cancer public proteomic datasets for biomarker discovery and validation

**DOI:** 10.1371/journal.pcbi.1011828

**Published:** 2024-01-22

**Authors:** Javier Robles, Ananth Prakash, Juan Antonio Vizcaíno, J. Ignacio Casal

**Affiliations:** 1 Department of Molecular Biomedicine, Centro de Investigaciones Biológicas Margarita Salas, Consejo Superior de Investigaciones Científicas, Madrid, Spain; 2 Protein Alternatives SL, Tres Cantos, Madrid, Spain; 3 European Molecular Biology Laboratory—European Bioinformatics Institute (EMBL-EBI), Wellcome Genome Campus, Hinxton, Cambridge, United Kingdom; University at Buffalo - The State University of New York, UNITED STATES

## Abstract

The cancer biomarker field has been an object of thorough investigation in the last decades. Despite this, colorectal cancer (CRC) heterogeneity makes it challenging to identify and validate effective prognostic biomarkers for patient classification according to outcome and treatment response. Although a massive amount of proteomics data has been deposited in public data repositories, this rich source of information is vastly underused. Here, we attempted to reuse public proteomics datasets with two main objectives: i) to generate hypotheses (detection of biomarkers) for their posterior/downstream validation, and (ii) to validate, using an orthogonal approach, a previously described biomarker panel. Twelve CRC public proteomics datasets (mostly from the PRIDE database) were re-analysed and integrated to create a landscape of protein expression. Samples from both solid and liquid biopsies were included in the reanalysis. Integrating this data with survival annotation data, we have validated *in silico* a six-gene signature for CRC classification at the protein level, and identified five new blood-detectable biomarkers (CD14, PPIA, MRC2, PRDX1, and TXNDC5) associated with CRC prognosis. The prognostic value of these blood-derived proteins was confirmed using additional public datasets, supporting their potential clinical value. As a conclusion, this proof-of-the-concept study demonstrates the value of re-using public proteomics datasets as the basis to create a useful resource for biomarker discovery and validation. The protein expression data has been made available in the public resource Expression Atlas.

## Author summary

The need for new prognostic biomarkers is one of the main topics of interest in CRC (Colorectal Cancer). A potential strategy to address this issue, which to the best of our knowledge has not been attempted so far, is the combination of different public proteomic studies generated from independent patient cohorts. Despite the abundance of available proteomics data, meta-analyses have only been conducted at the genomic and transcriptomic levels so far. In this study, we reanalyzed 12 mass spectrometry-based public proteomics datasets. In total, the combined dataset included 440 samples from 299 different patients, encompassing both solid and liquid biopsies. Consequently, we defined a proteomics landscape suitable for assessing protein expression in tumors and normal mucosa, its association with patient outcome, and its potential detection in liquid biopsies. Furthermore, as a proof-of-concept for the data reuse strategy, we demonstrated its capacity to validate an experimentally-based SEC6 gene signature at the protein level and to identify new blood-detectable biomarkers. The data generated in this study can be accessed by anyone since all the data have been made available in the Expression Atlas resource.

## Introduction

Colorectal cancer (CRC) is the third most common cancer worldwide, accounting for approximately 10% of all diagnosed cancers [1]. Notably, the most developed countries present the highest rates of incidence [2]. Regarding global mortality, CRC is the second leading cause of cancer-related deaths [3]. CRC is considered a highly heterogeneous disease [4], caused through various genetic and epigenetic mechanisms, including microsatellite instability or mutations in oncogenes such as APC, TP53, KRAS, and BRAF. The molecular heterogeneity leads to variability in the pathogenesis, outcome and treatment response [5]. Both clinical and molecular heterogeneity are essential challenges when facing CRC diagnosis and prognosis [6] and to face this heterogeneity, several molecular classifications have been proposed [7]. CRC diagnostic procedures have made significant advances in the last years based on the massive use of faecal occult blood screening tests, liquid biopsies, and the more invasive colonoscopy [8]. However, once the disease is detected, the current clinical stratification systems, based on the pathological staging, presents some limitations that fail to identify a relevant number of patients relapsing and/or developing metastases after surgical resection. For those reasons, it is especially crucial to identify prognostic biomarkers to categorize patients in stage II and III to prevent recurrence, and to identify those patients who should receive more intensive treatments [9]. Although, most of the approaches designed to address CRC diagnosis and prognosis have relied so far in samples from solid biopsies [4,10] obtained using highly invasive procedures, biomarkers detectable in liquid biopsies are a preferable option for predicting and monitoring patient outcome and response to chemotherapy [11]. Recent studies have demonstrated the importance of secreted proteins on tumor progression and metastasis development [12]. In summary, despite significant progress in CRC screening and diagnosis has been achieved [13], the problem of CRC prognosis and classification remains unresolved.

Most of the available high-throughput omics data coming from cancer patients are based on DNA and RNA sequencing information. This applies to both solid and liquid biopsies. Indeed, most of the attempts to classify CRC are based on transcriptomics data [4,10]. Nevertheless, proteins are most often the functional molecules that undertake the translation from genotype into the phenotype. In addition, protein-based techniques such as ELISA or immunohistochemistry have demonstrated to be useful tools with clinical relevance [14]. Mass spectrometry (MS) is the main high-throughput proteomics approach for providing quantitative measurements of protein abundance/expression [15]. Although publicly available genomics and transcriptomics datasets are more in number and scope, large relevant proteomics studies such as those performed by the Clinical Proteomic Tumor Analysis Consortium (CPTAC) [16] or other independent teams, have obtained MS-based protein expression information in CRC samples. Taken together, this protein expression data from different datasets can be considered as a robust source of information for biomarker discovery and confirmation.

Multiple proteomics datasets are freely available in public repositories. The PRIDE database (as the most popular resource in this context) [17], together with other open repositories (e.g. CPTAC portal [16], jPOST [18]) contain thousands of proteomics datasets. Public data in these resources coming from different sources can be reanalysed and integrated to obtain a global view and potentially discover new insights. Integrative meta-analyses have already demonstrated to be useful using genomics and transcriptomics data [19,20]. Regarding proteomics, quantitative reanalysis and integration of public data is emerging as a potent resource with multiple applications [21–25]. To our knowledge, no previous studies have addressed prognostic biomarker identification in CRC from this perspective of reusing public proteomics datasets. In the present study, we selected, reused, and integrated 12 public proteomics datasets containing samples from both solid and liquid biopsies of CRC patients. The reanalysed and processed data have been deposited in the Expression Atlas resource [26] to facilitate protein abundance data access and visualisation. In summary, we provide a CRC proteomic landscape with the capacity to validate previously reported prognostic biomarkers and to discover new sets of biomarkers, demonstrating the potential and value of reusing public proteomics datasets for biomarker discovery.

## Results

### Colorectal cancer proteomics datasets and integrative analysis

We queried PRIDE [17], jPOST [18] and the CPTAC [16] portal for CRC studies and selected 12 publicly available datasets for reanalysis (Table 1). These CRC datasets were divided in two groups, corresponding to secreted and solid tumor samples, respectively. The secretome group consisted of samples derived from blood, cell culture, extracellular vesicles, exosomes and interstitial fluid, whereas the solid tumor sample group consisted of samples derived from mucosa, adenoma (pre-malignant cellular masses), and tumor tissues. The secretome and solid tumor sample groups consisted of 7 and 5 datasets, respectively, and included paired healthy and cancer samples. The characteristics of the overall patient cohorts included in the datasets are shown in Table 2. Patient composition of the meta-analysis shows a non-biased, proportional distribution of the different sub-classifications of CRC, according to mutations, chromosomal stability, age and sex.

**Table 1:** List of the proteomics datasets used in this study.

Combined analysis	Tissues	Organs	Proteomics dataset identifier*	Expression Atlas identifier	Number of MS runs	Fractionation (Fractions per sample)	Number of samples	Number of patients	Number of protein groups†	Number of peptides†	Number of unique peptides†	Number of unique genes (canonical proteins) mapped†
**Solid samples**	Mucosa, colorectal adenoma, colorectal carcinoma	Colorectum	PXD001676 [58]	E-PROT-103	16	No	16	8	9,711	215,033	196,017	8,949
			PXD002137 [59]	E-PROT-104	192	Yes (6)	32	25				
			PXD014511 [60]	E-PROT-105	310	Yes (5)	62	52				
			PXD019504 [61]	E-PROT-106	74	No	74	37				
			CPTAC PDC000111 [31]	E-PROT-23	1425	Yes (15)	90	90				
**Total solid tumor**			**5 datasets**		**2,017**		**274**	**212**				
**Secreted samples**	Interstitial fluid, extracellular vesicles, blood serum, cell lines	Colorectum, liver, blood	PXD005709 [62]	E-PROT-100	36	Yes (3)	12	6	5,861	85,013	79,181	5,091
			PXD005693 [62]	E-PROT-101	15	No	15	8				
			PXD020454 [63]	E-PROT-102	45	Yes (3)	15					
			PXD010458 [64]	E-PROT-107	144	Yes (24)	6	16				
			JPST000867 [65]	E-PROT-108	68	No	36	17				
			PXD031556 [66]	E-PROT-109	79	No	79	40				
			PXD032899 [30]	E-PROT-110	54	Yes (3)	3					
**Total secreted**			**7 datasets**		**441**		**166**	**87**				
**TOTAL**			**12 datasets**		**2,458 MS runs**		**440 samples**	**299 patients**				

*Dataset identifiers starting with ‘PXD’ come from the PRIDE database, dataset JPST000867 is from jPOST and dataset PDC000111 is from CPTAC portal. Unique protein sample batches available in any given dataset are considered as individual samples. † Numbers after post-processing. The proteomics results in Expression Atlas can be accessed using the link: https://www.ebi.ac.uk/gxa/experiments/E-PROT-XXX/Results, where XX should be replaced by the E-PROT accession number shown in the table. The raw proteomics datasets in PRIDE can be accessed using the link: https://www.ebi.ac.uk/pride/archive/projects/PXDxxxxxx, where PXDxxxxxx should be replaced by the PRIDE dataset identifier shown in the table.

**Table 2:** Clinicopathological features of colorectal adenoma and adenocarcinoma patients included in the proteomic datasets.

Colorectal adenoma patients			Colorectal adenocarcinoma (CRC) patients		
Clinicopathological features		Cases (%)	Clinicopathological features		Cases (%)
**Age**	Age ≤ 65	56.41	**Age**	Age ≤ 65	27.66
	Age > 65	43.59		Age > 65	47.52
	Unspecified	0.00		Unspecified	26.95
**Gender**	Male	57.69	**Gender**	Male	37.59
	Female	41.03		Female	37.59
	Unspecified	0.00		Unspecified	26.95
**Site**	Colon	80.77	**Site**	Colon	50.35
	Rectum	17.95		Rectum	24.82
	Unspecified	0.00		Unspecified	26.95
**Subtype**	Conventional adenoma	19.23	**Stage**	I	11.35
	Sessile serrated adenoma	21.79		II	26.95
	Traditional serrated adenoma	15.38		III	19.86
	Unspecified	20.51		IV	9.22
					Unspecified	32.62
			**Microsatellite status**	MSS	58.87
				MSI	31.91
				Unknown	11.35
			***BRAF* status**	*BRAF* mut	5.67
				*BRAF* WT	58.16
				Unspecified	38.30
			***KRAS* status**	*KRAS* mut	29.08
				*KRAS* WT	34.75
				Unspecified	38.30
			***TP53* status**	*TP53* mut	24.82
				*TP53* WT	39.01
				Unspecified	38.30

**Fig 1:**
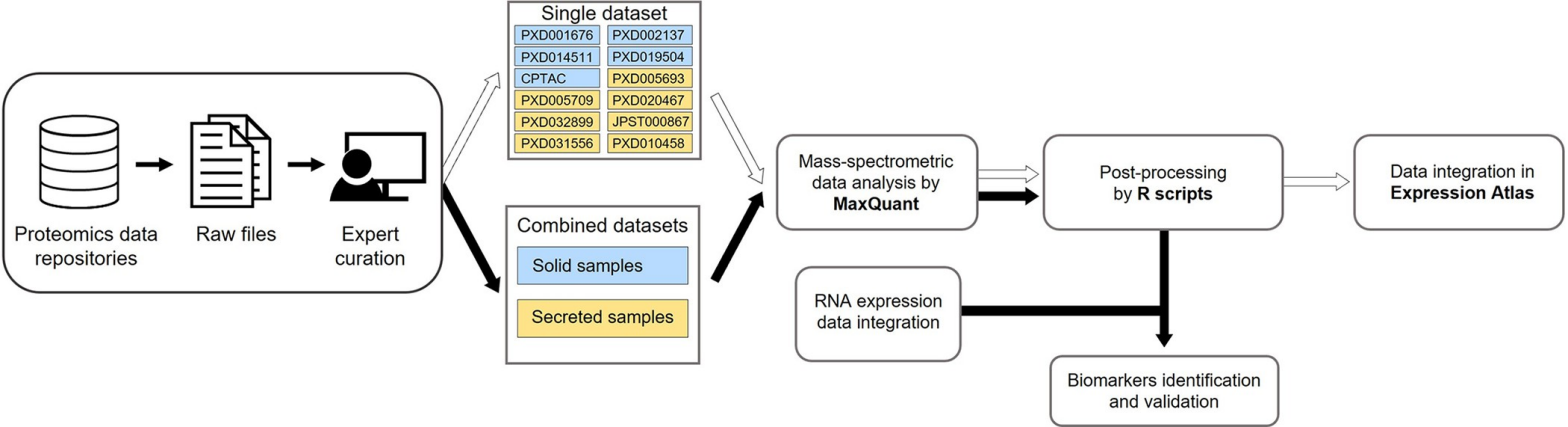
Scheme of the project design and the data reanalysis pipeline. Workflow of the selection, curation, reanalysis and integration of public proteomic dataset containing CRC samples.

We reanalysed each sample group separately by creating two batches (solid and secreted samples). Each batch consisted of all MS runs from all datasets that were part of a sample group. Protein identification and quantification analysis was performed using MaxQuant [27] version 2.1.0.0 on a high-performance computing Linux cluster. In total, the datasets contained 2,458 MS runs, covering 440 samples from 299 individuals. The number of protein groups, peptides, and unique peptides identified in the two batches of samples are shown in Table 1. The protein abundances calculated from individual datasets are available to view and download from Expression Atlas [28], along with their experimental parameters, sample metadata and summary of sample quality assessment after post-processing. The data reanalysis protocol is summarised in Fig 1 and explained in detail in S1 Fig.

### Protein abundance comparison in solid tumors and secreted samples

Protein abundances were calculated for the secretome and solid tumor samples using the intensity-based absolute quantification (iBAQ) method and normalised by the FOT (Fraction of Total) method (see Methods). We converted the normalised iBAQ abundance values of each sample into five abundance bins as previously described [25], for ease of comparison of abundances across samples and datasets. The abundance bins ranged from bin1 to bin5, denoting proteins with lowest to highest abundance, respectively. We mapped the protein identifiers within the protein groups to their respective parent gene names equivalent to ‘canonical proteins’ in UniProt (see ‘Methods‘) and, hence, when describing protein abundances in the manuscript we refer to the abundance of the ‘canonical proteins’.

### Protein identification in solid tumor samples

**Fig 2:**
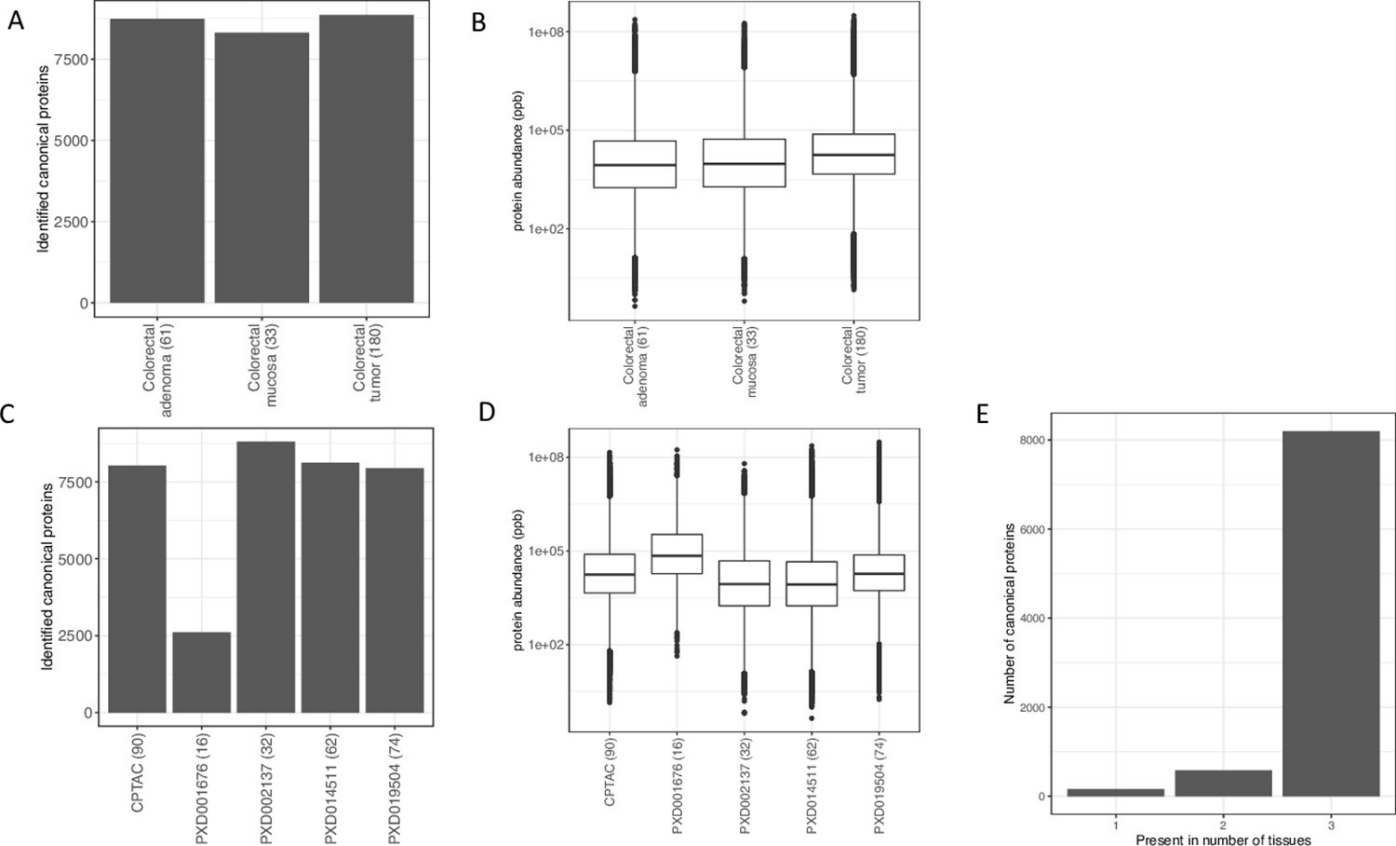
Colorectal cancer (CRC) solid samples. (A) Number of canonical proteins identified across different solid tumor samples. (B) Range of normalised iBAQ protein abundances across different samples. (C) Number of canonical proteins identified across different datasets. (D) Range of normalised iBAQ protein abundances across different datasets. (E) Number of canonical proteins identified across either one, two or three of the different solid samples subgroups (mucosa, adenoma and tumor). The number within the parenthesis indicate the number of samples.

Across all tumor samples we identified a total of 9,711 protein groups, which we mapped to 8,949 parent genes (canonical proteins). We identified similar numbers of canonical proteins across samples from colon mucosa, colon adenoma, which are pre-cancerous cell masses, and colorectal tumor samples (Fig 2A). The dynamic range of protein abundances was similar across tissue samples (Fig 2B). Across datasets, PXD001676 had the lowest number (29.1%) of identified canonical proteins (Fig 2C). However, dataset PXD001676 showed a higher median protein abundance relative to the other datasets (Fig 2D), probably due to the fewer number of canonical proteins identified in this dataset. We found that the large majority of canonical proteins was simultaneously present in the three types of samples: mucosa, adenoma and tumor (Fig 2E). Compared to the secreted samples, the correlation of protein abundance among tumor tissues was relatively low (S2 Fig). The highest correlation was observed in adenoma samples (median R^2^ = 0.58) and the lowest correlation was among mucosa samples (median R^2^ = 0.49).

### Protein identification in secreted samples

**Fig 3:**
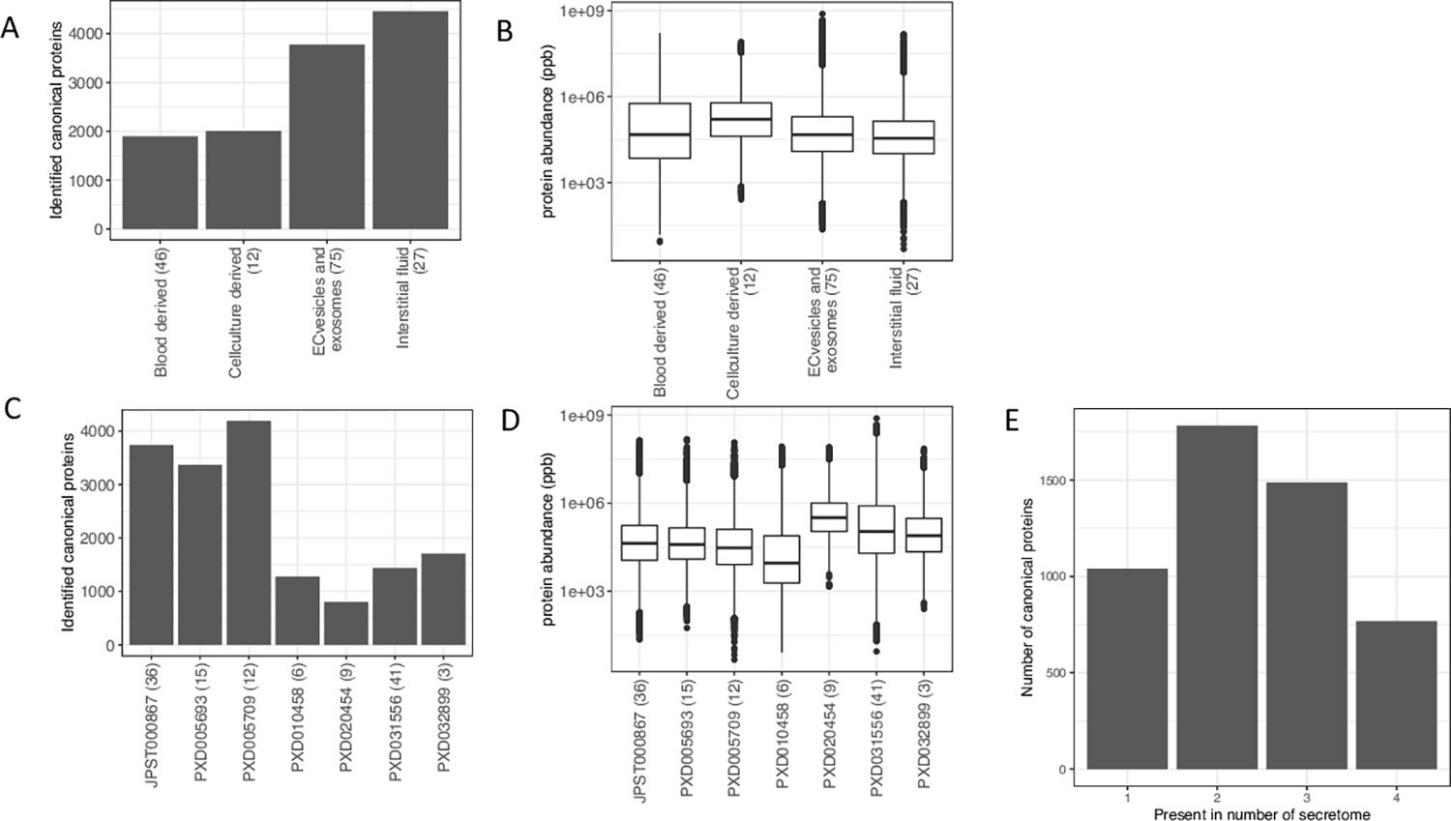
Colorectal cancer secreted samples. (A) Number of canonical proteins identified across different secreted samples. (B) Range of normalised iBAQ protein abundances across different samples. (C) Number of canonical proteins identified across different datasets. (D) Range of normalised iBAQ protein abundances across different datasets. (E) Number of canonical proteins identified across either one, two, three or four of the different secreted samples subgroups. The number within the parenthesis indicate the number of samples.

We obtained 5,861 protein groups from the secreted samples, which were mapped to 5,091 parent genes (canonical proteins). We identified a large number of proteins in samples from interstitial fluid (87.5%) and extracellular vesicles and exosomes (74.2%), while comparatively less proteins were identified in blood-derived (37.2%) and cell culture samples (39.4%) (Fig 3A). The dynamic range of protein abundances across secreted samples and datasets is shown (Fig 3B and 3D). Among datasets, the largest numbers of canonical proteins were identified in datasets PXD005709, JPST000867 and PXD005693 (Fig 3C). The median protein abundance was highest in cell culture-derived samples compared to other samples, and, similarly, dataset PXD020454 had a higher median protein abundance, likely due to the low number of canonical proteins identified in this dataset. The number of canonical proteins identified across all subgroups was relatively low, likely influenced by the unequal distribution of identifications among the subgroups. Indeed, the majority of canonical proteins were exclusively present in either two or three of the subgroups (Fig 3E). We used the binned protein abundances to compare the correlation of protein abundances across secreted samples. We also observed a good correlation and clustering of samples at the heatmap (S3 Fig). The highest correlation in protein expression was observed among blood-derived samples (median R^2^ = 0.80) and the lowest correlation was observed among cell culture-derived samples (median R^2^ = 0.32), which confirms the high heterogeneity of cancer cell lines.

### Proteins in secreted fractions mirror tumor alterations

**Fig 4:**
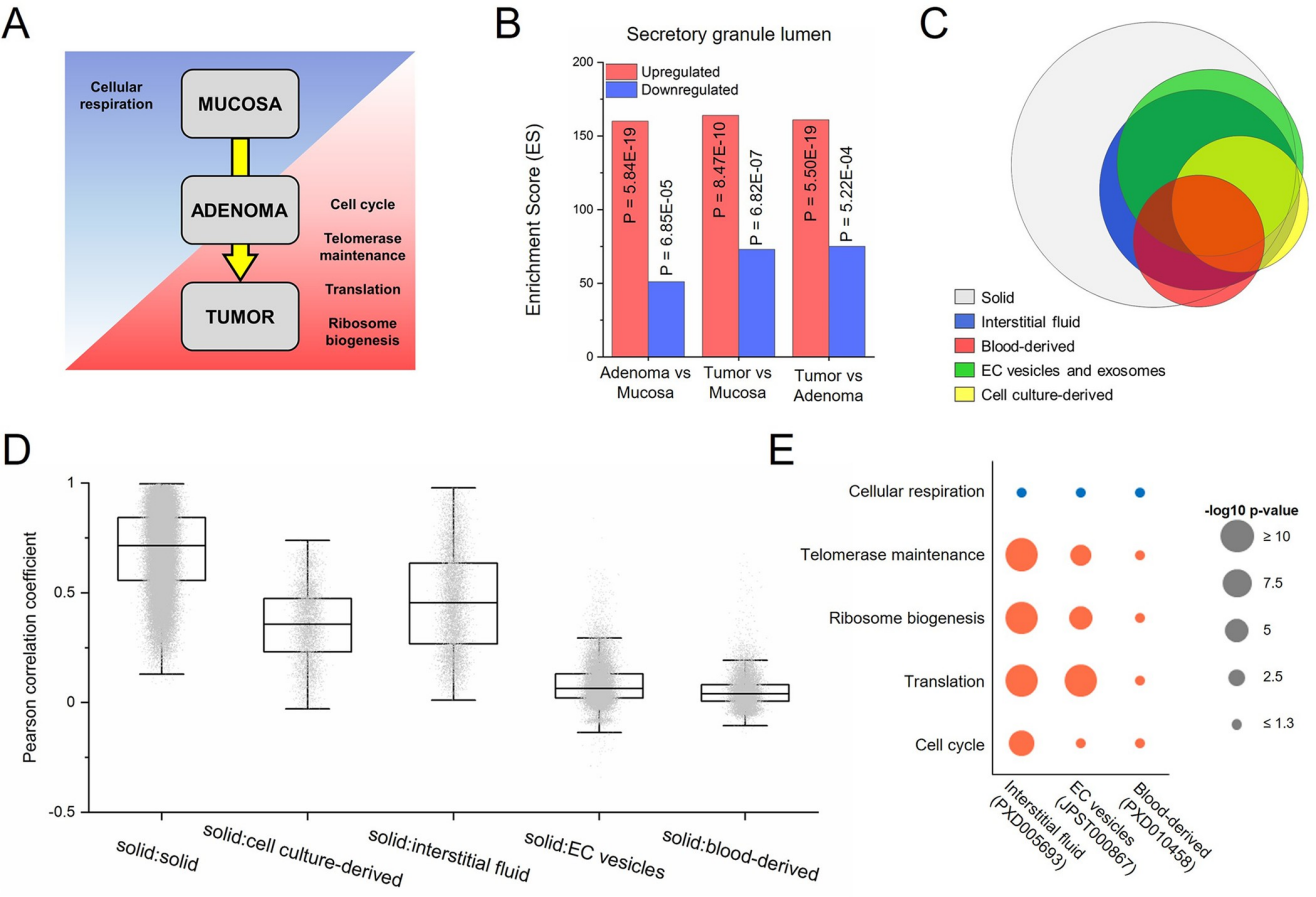
Analysis of GO enrichment and concordance between solid and secreted samples. (A) Plot summary illustrating the most altered GO terms in the “biological process” category in solid samples. (B) Enrichment in ‘Secretory granule lumen’ (GO0034774) category of upregulated and downregulated proteins when comparing mucosa, adenoma and tumor samples. (C) Venn diagram representing the detected canonical proteins in solid and secreted samples. (D) Boxplot showing the correlation between solid samples and each of the subgroups of secreted samples. (E) Significance of enrichment in GO Biological Process categories of altered proteins (tumor vs normal mucosa) in different secreted subgroups.

To better understand the biological alterations during CRC tumorigenesis, following the pipeline described in ‘Methods’, we identified differentially-expressed proteins comparing normal mucosa, adenomas and CRC tumor samples. First, we performed Gene Ontology (GO) enrichment analysis to compare the three types of solid samples (Fig 4A). The GO categories significantly enhanced in tumor samples when compared with the normal mucosa and adenoma were ‘cell cycle’ (GO0007049), ‘telomerase maintenance’ (GO0000723), ‘translation’ (GO0006412), and ‘ribosome biogenesis’ (GO0042254). These categories are related to an increased proliferation in the tumors. Conversely, the most significant biological process decreased during tumor progression was ‘cellular respiration’ (GO0045333), probably due to the Warburg effect [29]. When analysing the GO Cellular Component category, there was a significant enrichment of the secreted fraction ‘secretory granule lumen’ term (GO0034774) (Fig 4B). This enrichment was found to be higher in the overexpressed proteins when comparing “Adenoma vs Mucosa”, “Tumor vs Mucosa” and “Tumor vs Adenoma” indicating that the secreted fraction increases through tumor progression in CRC, due to the high intestinal secretory component.

Given the relevance of the secreted fraction in CRC progression^11^, the level of agreement in protein expression between solid tumor samples and secreted fractions was investigated. Most of the proteins quantified in the secreted fractions were also present in the solid samples (Fig 4C). To determine concordance, the Pearson correlation coefficient (r) values between solid tumor samples and each secreted source were calculated (Fig 4D). On secreted fractions, the interstitial fluid and cell culture-derived samples showed greater correlations with the tumor samples than with extracellular vesicles and blood-derived samples. Then, we tested whether the secreted fractions displayed similar biological alterations to those found between tumor and mucosa in solid samples (Fig 4E). Interstitial fluid, and EC vesicle samples to a lower extent, were able to replicate the changes observed in the solid tumors, where blood-derived samples were not. In addition, correlation between the fold-changes in tumor *vs* mucosa was analysed. Whereas EC vesicles and interstitial fluid samples presented a high correlation with solid samples (R = 0.48 and R = 0.45, respectively), blood-derived samples did not (R = 0.05) (S4A Fig). Of note it is the high correlation found between EC vesicles and interstitial fluid samples (R = 0.80).

We defined as “enriched proteins” those canonical proteins exclusively or highly expressed in just one of the secreted subgroups (S4B Fig). Then, “enriched proteins” of each secreted source were analysed to identify the overrepresented functions in each subgroup (S4C Fig). Interstitial fluid proteins were particularly relevant in the study of ‘cellular respiration’ (GO0045333) and ‘translation’ (GO0006412), while EC vesicles were the best option for the ‘cell cycle’ (GO0007049). “Enriched proteins” from cell culture supernatants and blood-derived samples were mainly involved in ‘wound healing’ (GO0042060) and ‘blood coagulation’ (GO0007596). Moreover, in all subgroups, “enriched proteins” were significantly associated with GO Cellular Component categories corresponding to secreted fractions, such as ‘EC vesicle’ (GO1903561) or ‘EC region’ (GO0005576) (S4D Fig). Overall, these analyses suggest that secreted fractions represent a suitable model for the study of protein expression in CRC, as they show a remarkable correlation with solid tumor alterations.

### Concordance between mRNA and protein-based prognostic biomarkers in colorectal cancer

**Fig 5:**
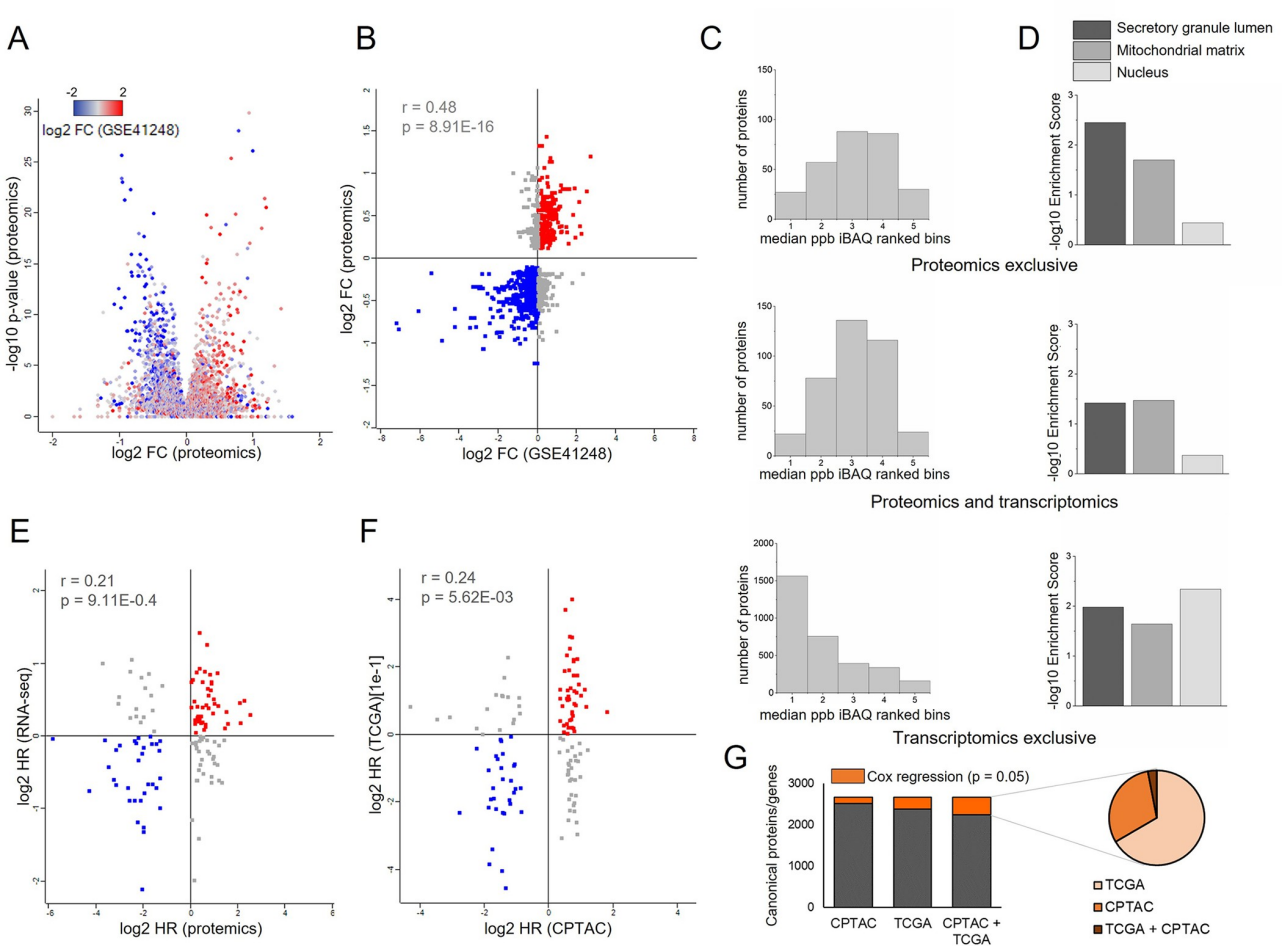
Correlation between transcriptomics and proteomics analysis. (A) Volcano plot distribution of the proteomics data. Fold change (Tumor/mucosa) is represented. Dots are labelled according to the transcriptomic fold change. (B) Scatter plot of significantly altered canonical proteins. Pearson’s coefficient is shown. (C) Histogram distribution of protein expression levels (ranked bins) and (D) Cellular Component analysis of proteins or genes significantly altered between normal mucosa and tumor in proteomics and/or transcriptomics. More relevant categories were selected. (E) Correlation of hazard ratios obtained using proteomics data and RNA-seq data from CPTAC. (F) Correlation of hazard ratios obtained using proteomics data (CPTAC) and RNA-seq data (TCGA). Only significantly prognosis-associated proteins are shown. Pearson correlation coefficient (r) is indicated. (G) Portion of the canonical proteins significantly associated with survival according to proteomics (CPTAC) and/or transcriptomics (TCGA) data. Significance was calculated by Cox regression analysis. Chart pie of all the significant associated mRNA (TCGA) and proteins (CPTAC).

To the best of our knowledge, there has been limited investigation into the large-scale concordance between gene expression- and protein expression-based biomarkers. To investigate which protein alterations are present at the gene expression level, a comparison between transcriptomics and proteomics fold-changes in tumor and normal mucosa samples was performed. Proteomics data from the solid samples batch was used after normalization into ranked bins. Transcriptomics data from primary tumor and normal mucosa samples was obtained from the GSE41258 public dataset from GEO (see –Methods–). Our analysis showed an excellent agreement between transcriptomics and proteomics alterations, as shown in a volcano plot representing all the quantified proteins (Fig 5A) and a scatter plot presenting only the significantly altered proteins (Fig 5B). Despite the overall concordance, we still found some disagreements. To explain these discrepancies, we analysed separately proteins that were significantly altered in either one or both analyses. Firstly, we examined the protein expression levels in tumor samples. Proteins significantly altered between tumor and mucosa in the proteomic analysis showed higher expression ranked values than the proteins corresponding to the altered genes detected in transcriptomics (Fig 5C). These results suggest that some of the exclusively-detected transcriptomic alterations were not detected using proteomics because expression values were too low to find differences between conditions. To examine the GO Cellular Component differences, three significant representative locations were identified: ‘Secretory granule lumen’ (GO0034774), ‘Mitochondrial matrix’ (GO0005759) and ‘Nucleus’ (GO0005634). We obtained different location profiles depending on the technique used for the detection. Proteomics-derived proteins were mainly enriched in secreted proteins, whereas nuclear genes were the most enriched location in transcriptomic-exclusive genes (Fig 5D). These findings might be explained by the relatively low expression of transcription, splicing and other nuclear factors.

Next, we evaluated the predictive potential of protein expression and its concordance with the RNA-seq values. Firstly, we selected proteins quantified in the CPTAC COADREAD dataset and the corresponding mRNA data in the CPTAC samples within the TCGA COADREAD datasets. CPTAC was selected because is the only CRC public proteomics dataset that contains patient survival data. In addition, it is the only dataset in which samples have been analysed both by proteomics and RNA-seq. Then, Cox regression analysis was performed to identify proteins or genes associated with survival. Significant proteins in the Cox regression analysis were plotted in scatter plots representing the hazard ratios (HR). A relatively low correlation was observed when comparing proteomics CPTAC data with CPTAC and TCGA COADREAD RNA-seq datasets (Fig 5E and 5F). Then, the ratio of significant proteins (CPTAC) and genes (TCGA) was determined. In the CPTAC dataset, from the initial 2,661 analysed proteins, 142 (5.3%) were significantly associated with prognosis, whereas 285 (10.7%) genes were significant in the analysis of the corresponding genes in the TCGA dataset (Fig 5G). A pie chart showed that 130 (30.4%) of the significant potential biomarkers were exclusively detected by proteomics (Fig 5G). This suggests that potential biomarkers identified by proteomics may go unnoticed when analysing mRNA values, and vice versa. Therefore, when searching for biomarkers on a large scale, it is optimal to integrate proteomics and transcriptomics data to attain a more comprehensive biomarker panel.

### Validation of experimentally-derived biomarkers using public proteomics datasets

**Fig 6:**
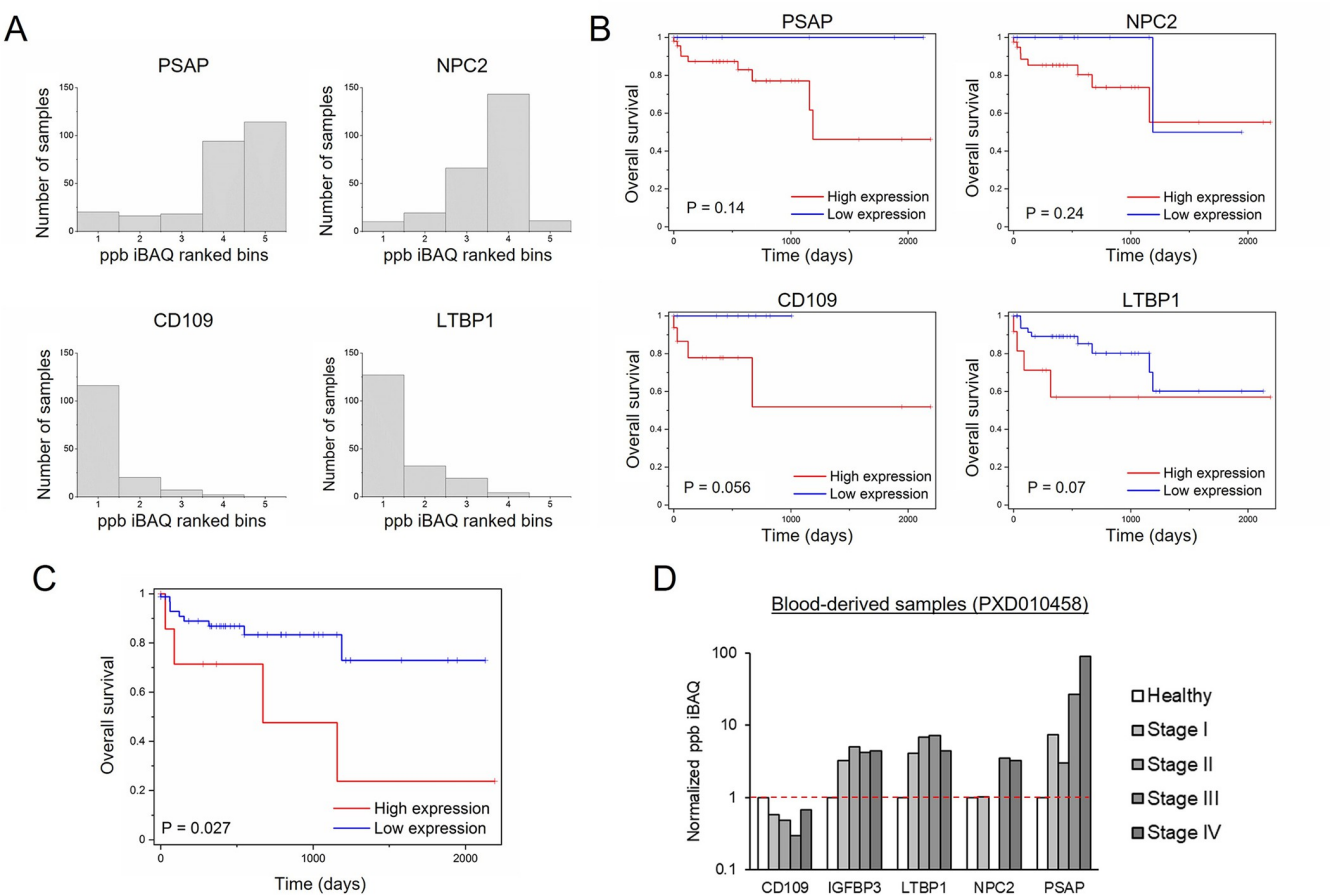
Validation at the proteomic level of the experimentally-based signature SEC6. (A) Histogram distribution of the expression of the SEC6 proteins detected in more than 50% of the tumor samples using CPTAC dataset (iBAQ ranked bins are used). (B) Kaplan–Meier analysis of high- and low-expression patients in stage II and III patients. P values were obtained by log-rank test. (C) Kaplan–Meier analysis of high- and low-expression patients. Mean protein expression of CD109, LTPB1, NPC2 and PSAP was used for classification. P values were obtained by log-rank test. (D) SEC6 proteins distribution across blood-derived samples.

As an initial proof-of-concept, we applied our strategy to the validation of a recently reported prognostic and predictive signatures for CRC based on the expression of six genes (BMP1, CD109, IGFBP3, LTBP1, NPC2, PSAP), which were identified through proteomics analysis of the secretoma of metastatic cancer cells and validated in transcriptomics datasets [30]. Protein descriptions and their UniProt accession numbers can be found in S1 Table. Therefore, we determined whether this signature could also be detected in the proteomic datasets, according to our in silico reanalysis strategy. At the proteomic level, four out of six proteins, corresponding to the genes CD109, LTBP1, NPC2 and PSAP, were detected in more than 50% of the solid tumor samples included in the CPTAC dataset (Fig 6A), and were considered for their association with survival using Kaplan-Meier analysis (Fig 6B). A clear trend was observed, since patients with high expression in all four proteins showed lower overall survival rate. As only four proteins were quantified using proteomics datasets, instead of the original calculation of the risk score based on an algorithm including the six genes, a simpler approach based on the mean of the four available proteins was performed to classify patients in high and low risk. A previous normalization (division by mean) was done to avoid the high differences between protein levels. Kaplan-Meier analysis showed a significant association of the combined protein expression with poor survival (Fig 6C). Furthermore, as SEC6 are secreted proteins, their presence in blood-derived samples was analysed. In this case, all the proteins, except BMP1, were detected in blood-derived samples, and four of them (IGFBP3, LTBP1, NPC2 and PSAP) were highly increased in CRC patients (Fig 6D). The high levels found in the blood of colorectal patients agree with the previous detection in the secretome and represent a promising added value to the signature.

### Discovery of new secreted biomarkers using public proteomics datasets

**Fig 7:**
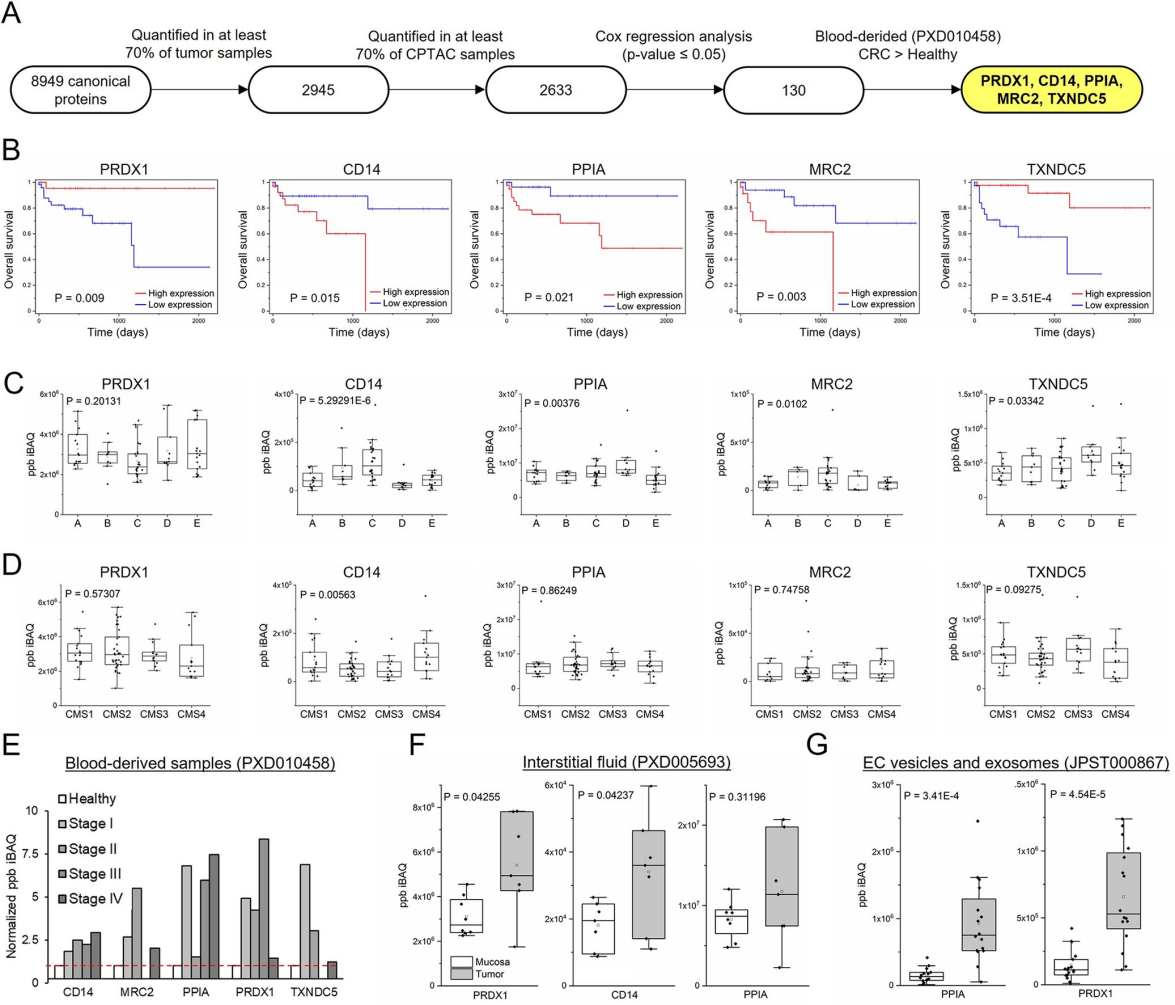
Identification of blood-detectable prognostic biomarkers. (A) Flow-chart representation of sequential prognostic biomarkers selection. (B) Kaplan–Meier analysis of high- and low-expression patients. P values were obtained by log-rank test. ((C) Distribution of the protein expression (ppb) according to the proteomic subtypes’ classification [59]. (D) Distribution of the protein expression (ppb) according to the CMS classifier. (E) CD14, MRC2, PPIA, PRDX1,TXNDC5 distribution across blood-derived samples. (F) PRDX1, CD14 and PPIA distribution across interstitial fluid from tumor and mucosa according to the PXD005693 dataset. (G), PPIA and PRDX1distribution across EC vesicles from tumor and adjacent tissue according to the JPST000867 dataset.

Finally, we searched for new proteomics-derived prognostic biomarkers, as an additional proof-of-concept for our hypothesis. To achieve this, we followed the indicated workflow (Fig 7A). Proteins quantified in 70% of all solid tumor samples and, then, at least in 70% of CPTAC samples were selected. Association with survival was determined using Cox regression analysis. We identified 130 proteins significantly associated with prognosis (Fig 5G). For potential biomarker detection in liquid biopsies, selection was restricted to those proteins overexpressed in blood samples from CRC patients. Five candidate biomarkers (CD14, MRC2, PPIA, PRDX1, and TXNDC5) were obtained. Whereas PRDX1 and TXNDC5 were biomarkers of good prognosis, CD14, PPIA and MRC2 were associated with poor prognosis (Fig 7B). Protein descriptions and UniProt accession numbers can be found in S2 Table. Kaplan-Meier analysis confirmed a prognostic value when considering all patients (Fig 7B) or only patients in stages II and III (S5A Fig), where clinical treatments require more complex decisions. To test if these potential biomarkers could be also used in combination, patients were classified in high or low risk according to the expression levels of each protein (S5B Fig). The significance of the classification increased when we considered as high-risk patients those in whom at least three of the five proteins classified them as high risk. Kaplan-Meier analysis was significant for either all the patients or stage II and III patients (S5C Fig).

Next, the prognostic efficiency of these proteins was evaluated at the transcriptomic level using the TCGA RNA-seq database and the best cut-off Kaplan-Meier significance. All the corresponding genes were significant, except for TXNDC5, which was close to significance (6A Fig). When a more restrictive Cox regression test was performed, these biomarkers showed significance using proteomics data (CPTAC), but not at the mRNA level (TCGA data), indicating that the signal is stronger at the protein level (S6B Fig). Additionally, the prognostic value of these biomarkers was evaluated in eight independent transcriptomics datasets by using a log-rank test (S6C Fig). Although the biomarkers were not significantly associated with outcome in all the independent datasets, the signal was stronger when combining all the cohorts.

To further explore the clinical value and potential applications to patient stratification of these biomarkers, we explored the expression in the different CRC subtypes, according to proteomic (Fig 7C) and transcriptomic (Fig 7D) classifications. There was a significant statistical association of the 5 biomarkers with the proteomic classification [31], except for PRDX1. CD14 and MRC2 were more abundantly expressed in the subtype C associated with poor outcome, whereas TXNDC5 showed higher expression in subtype D (Fig 7C). In contrast, the significance of the association with the transcriptomic-derived consensus molecular subtypes (CMS) was lower, except for CD14, which showed higher expression in the CMS4 and CMS1 subtypes (associated with poor prognosis), and TXNDC5 that showed more expression in the good prognosis CMS3 subtype. Therefore, proteomic markers correlated better with proteomic-defined subtypes than with transcriptomics-identified ones. In any case, CD14 and TXNDC5 showed strong association with poor and good prognosis, respectively, in a variety of independent datasets, endorsing their value as prognostic biomarkers.

Regarding location, these biomarkers showed higher expression in serum from CRC patients when compared to healthy controls (Fig 7E). PRDX1, CD14 and PPIA were also detected and upregulated in interstitial fluid samples (Fig 7F). Finally, PPIA and PRDX1 were both detected and overexpressed in tumor-derived extracellular vesicles (Fig 7G). In conclusion, our meta-analysis supports a novel approach for the identification of novel protein biomarkers suitable for detection in liquid biopsies. Moreover, our data indicate that, for some biomarkers, proteomics detection may outperform transcriptomics analysis.

### Tissue expression and functional analysis of the biomarkers panel

To further explore the biological significance of these candidates in CRC tumor samples, immunohistochemistry images were retrieved from the Human Protein Atlas (HPA) [32] (S7A Fig). While PPIA, PRDX1, and TXNDC5 showed abundant expression in most samples, the detection of MRC2 and CD14 was lower (S7B Fig), likely because they are mainly stromal proteins expressed by fibroblasts and macrophages, respectively. In any case, a significant prognostic value of PPIA (p = 0.076), PRDX1 (p = 0.021), and TXNDC5 (p = 0.033) was confirmed using the HPA dataset.

**Fig 8:**
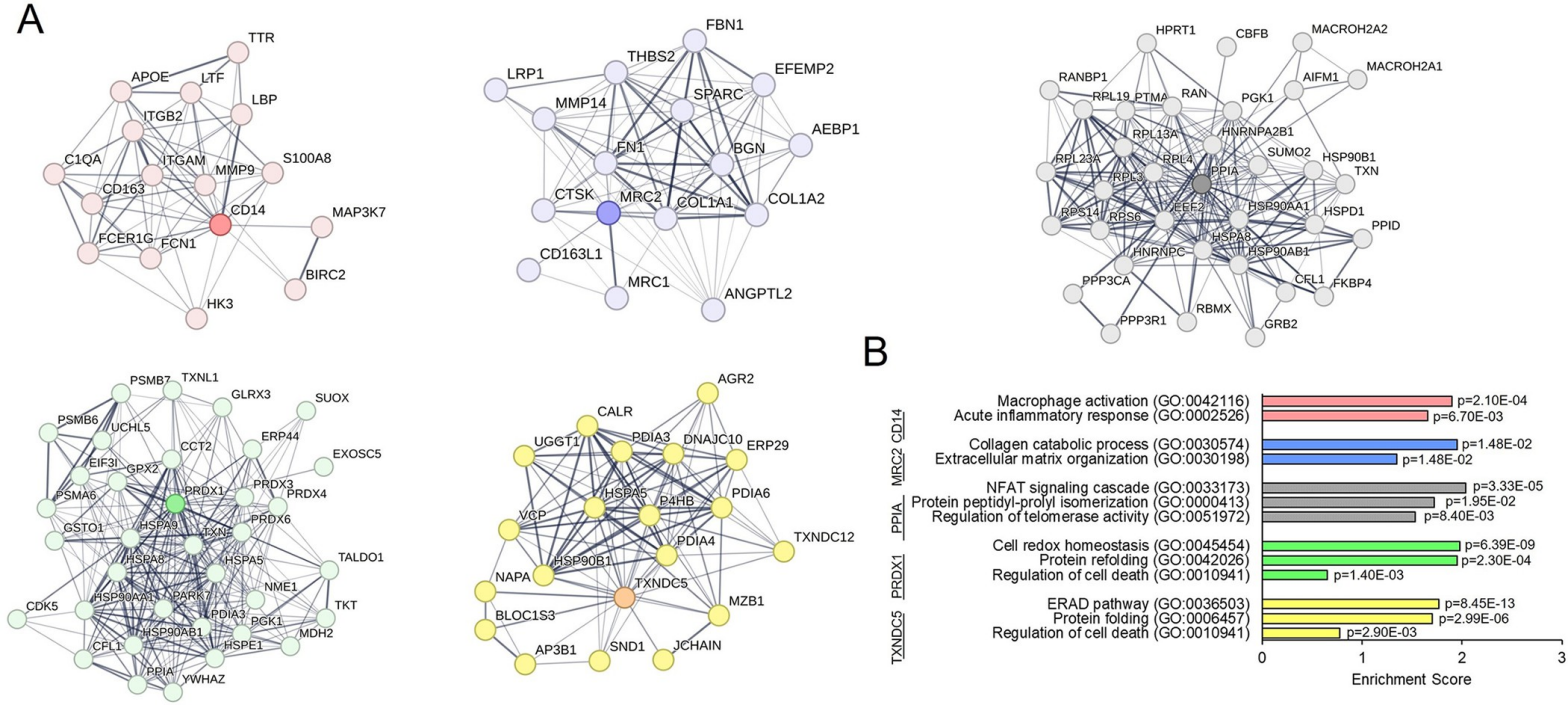
Functional analysis of the identified biomarkers. (A) STRING protein-protein interaction network of the five biomarkers. Proteins with at least medium confidence interaction (score>0.4) and a significant correlation (p<0.01 according to a Pearson correlation) with the corresponding biomarker were selected. Protein expression (ppb) resulting from the solid samples meta-analysis were used for calculating the correlations. (B) Functional enrichments (Biological Process) of the networks according to STRING.

Next, we carried out a functional analysis in order to investigate the potential mechanistic basis of these biomarkers. To identify the proteins associated with the discovered biomarkers, we firstly selected those proteins that presented a high Pearson correlation (p<0.01) with each biomarker when analysing the solid tumor samples. Then, proteins with at least one interaction with the biomarkers were analysed using STRING [33] to obtain the interactome of the biomarkers (Fig 8A). An interactome analysis based on GO revealed major associations with diverse biological functions (Fig 8B). CD14 was mainly associated to inflammatory processes as “macrophage inflammation” and “acute inflammatory response”. MRC2 was associated with collagen processing and “extracellular matrix organization”. PPIA showed interactions with proteins form the protein peptidyl-prolyl isomerization, and was involved in functions such as NFAT signalling or telomerase activity. Finally, the markers associated with good prognosis, PRDX1 and TXNDC5, were related with similar pathways, “protein refolding” and “regulation of cell death”, suggesting that, under cancer-related stress conditions, such as redox unbalance or abnormal protein folding, these proteins would suppress the cell cycle or participate in the cell death.

## Discussion

Our study provides a direct demonstration of the value of reusing public proteomics datasets for biomarker identification and validation. In this report, we reanalysed and integrated twelve public proteomic datasets from CRC samples (containing not only solid tumors but also liquid biopsy samples), to confirm the prognostic potential of a gene expression-based signature (SEC6) at the proteomic level [30], but also for the discovery of new candidate biomarkers capable of predicting patient outcome (CD14, MRC2, PPIA, PRDX1, TXNDC5). To the best of our knowledge, this is the first time that public proteomics datasets have been reused and focused in the detection and validation of biomarkers.

Large-scale genomics and transcriptomics studies have been conducted for biomarker discovery. Although proteomics meta-analysis studies are not yet as common as genomics and transcriptomics studies, they are becoming increasingly popular. However, manual curation is an essential step to provide the optimal level of metadata annotation, and to potentially identify biomarkers from data mining alone [34] A few proteomics meta-analysis studies focusing on human malignancies, such as cancer [22] or Alzheimer’s disease [35], have already been published. Proteomics meta-analysis studies have some limitations, mainly related with the heterogeneity associated to proteomics data workflows and the resulting datasets. To achieve a better data integration and comparability, our study was restricted to label-free DDA (Data Dependent Acquisition) quantification studies. However, as original data were acquired in different experimental conditions, the presence of batch effects was expected. To minimize these batch effects, we used a rank-binned normalization [24,25]. Despite the mentioned limitations, meta-analysis studies have notable advantages. The main benefit is the possibility of reusing large amounts of data that, otherwise, would require unnecessary, time-consuming and expensive resources to be acquired again by MS. In addition, integrating multiple studies enables to examine a diverse range of biological conditions, which is difficult to achieve experimentally and can expand the number of proteins of interest that are detected. Additionally, all data in our study were analysed and processed using the same analysis pipeline and search database, which minimizes downstream variability and enables comparison and integration of datasets from different origin. Finally, in our view, compliance with open data sharing practices is essential [36,37]. Therefore, the protein abundance results have been made available in the Expression Atlas resource and can be accessed and visualised there (Table 1).

The identification of novel potential biomarkers in CRC is essential for personalized medicine, enabling the best therapeutic option for each patient. An appropriate categorization of stage II and III patients, considering the diversity within CRC, can allow consistent monitoring and chemotherapy treatment regardless of surgical approaches. With this objective in mind, a gene signature called SEC6 [30] was reported for the calculation of a risk score based on the RNA levels of six genes, predicting the patient outcome and their response to chemotherapy. Although the survival metadata associated with proteomic datasets is much smaller than those associated with genomics and transcriptomics data, we confirmed the prognostic value for four out of six of the proteins. Two of them, BMP1 and IGFBP3, were not detected by proteomics in most of the solid tumor samples. However, IGFBP3 was found as the most abundant protein in blood-derived samples. The fact that five out of these six proteins can be detected in blood greatly increases the potential of this signature for patient risk and response monitoring.

Our proteomic “re-use” strategy also enabled the identification of novel prognostic biomarkers in CRC using proteomics data alone. The CPTAC dataset was essential in this process, as it is the only one that contains survival data. This dataset has been frequently used for confirmation of previously identified prognostic biomarkers, usually through genomics or transcriptomics approaches [38,39]. In our study, we performed a large-scale analysis identifying all the proteins associated with prognosis according to CPTAC. In contrast to common practice, we validated our proteomics discoveries using RNA-seq data to confirm their prognostic potential. We identified five potential prognostic biomarkers (CD14, PPIA, MRC2, PRDX1, and TXNDC5). CD14 is a macrophage-associated marker that has been associated with unfavourable prognosis in CRC [40]. Peptidylprolyl isomerase A (PPIA), a.k.a. as cyclophilin A, has been related with ERK1/2 phosphorylation and NF-κB activation [41], gastrin cancer serum biomarker [42] and poor prognosis in hepatocellular carcinoma [43]. MRC2 (Mannose Receptor C Type 2) is a mannose receptor whose expression is upregulated and associated with prognosis in some types of cancer such as glioblastoma, bladder, ovarian, and renal cancer [44]. GO analysis of these three unfavourable markers showed association with inflammatory pathways and extracellular matrix reorganization, which are processes linked to invasion and progression. On the other hand, for PRDX1 and TXNDC5, identified as favourable prognostic biomarkers, the GO analysis showed association with cell death regulation. PRDX1 is a peroxiredoxin, an enzyme involved in regulating reactive oxygen species. Although its mechanism of action is uncertain, it has been shown to prevent metastasis and angiogenesis [45]. PRDX1 depletion promoted the expression of pro-inflammatory cytokines in CRC [46]. Finally, TXNDC5 is a disulphide isomerase (PDI) that catalyses protein folding and thiol-disulphide interchange reactions. TXNDC5 promotes survival and proliferation by inducing HIF-1α in hypoxic situations [47]. Although upregulated in different tumours, its role in cancer progression remains unclear [48]

In summary, the CRC protein abundance/expression landscape resulting from the reanalysis of twelve public datasets constitutes a rich source of information for biomarker discovery and validation. To the best of our knowledge, this is the first time that a meta-analysis of public proteomics datasets has been used as the basis for biomarker discovery and validation. Additionally, we have demonstrated its value to perform, in a relatively short period of time, the validation of previously described biomarkers and the identification of new biomarker panels with potential clinical utility.

## Methods

### Dataset selection

MS-based proteomics data from studies of human colorectal cancer were selected for reanalysis from public repositories included in ProteomeXchange such as PRIDE and jPOST, and from the CPTAC data portal. These databases were queried for human CRC and the resulting hits were filtered based on the following criteria- i) label-free DDA studies, where no post-translational modification (PTM)-enrichment had been performed; ii) experiments performed on Thermo Fisher Scientific instruments (LTQ Orbitrap, LTQ Orbitrap Elite, LTQ Orbitrap Velos, LTQ Orbitrap XL ETD, LTQ-Orbitrap XL ETD, Orbitrap Fusion and Q-Exactive); and iii) availability of detailed sample metadata in the original publication, or after contacting the original submitters. As a result, 10 datasets from PRIDE, one dataset each from jPOST and one from the CPTAC data portal were downloaded. The details of these datasets are available in Table 1. It is important to highlight that, although a small number of additional public datasets generated using other proteomics approaches were available, the 12 chosen datasets represented the vast majority of the relevant CRC public proteomics datasets. All datasets were manually curated and the corresponding information was encoded in a SDRF (Sample Data Relationship File), linking the MS raw data to the biological conditions.

### Proteomics raw data processing

Proteomics datasets of secretome and tumor samples were analysed in two batches separately. Peptide/protein identification and protein quantification was performed using MaxQuant [27] (version 2.1.0.0) on a high-performance Linux computing cluster. The input parameters for each dataset such as MS1 and MS2 tolerances, digestive enzymes, fixed and variable modifications were set as described in their respective publications together with two missed cleavage sites. PSM (Peptide Spectrum Match) and protein FDR (False Discovery Rate) levels were set at 1%. Other MaxQuant parameter settings were left as default: maximum number of modifications per peptide: 5, minimum peptide length: 7, maximum peptide mass: 4,600 Da. For match between runs, the minimum match time window was set to 0.7 seconds and the minimum retention time alignment window was set to 20 seconds. The UniProt Human Reference Proteome (one protein sequence per gene set (*Homo sapiens*, UniProt, Sept. 2020. 20,601 sequences) was used as the target sequence database. The inbuilt MaxQuant contaminant database was also used, and the decoy database were generated by MaxQuant at the time of the analysis (on-the-fly) by reversing the input database sequences after the respective enzymatic cleavage.

### Post-processing

MaxQuant results for each batch were processed downstream to remove potential contaminants, decoys and protein groups which had fewer than 2 PSMs. The protein intensities were normalised using the FOT method as mentioned [25], wherein each protein iBAQ intensity value is scaled to the total amount of signal in a given MS run and transformed to parts per billion (ppb).


ppb_iBAQi=(iBAQi∑i=1niBAQi)x1,000,000,000

The bioconductor package ‘mygene’ [49] was used to assign Ensembl gene identifiers/annotations to the protein groups by mapping the ‘majority protein identifiers’ within each protein group. This step is required for integration into Expression Atlas. Briefly, from the MaxQuant output file ‘proteinGroups.txt’, the UniProt protein accessions within each protein group in the ‘majority protein identifiers’ columns were individually queried using the ‘queryMany’ function in the ‘mygene’ package to obtain their respective Ensembl gene symbols and gene identifiers. The protein groups, whose protein identifiers were mapped to multiple Ensembl gene symbols/IDs, were not used for further downstream analysis. In those cases, where two or more protein groups mapped to the same Ensembl gene symbol/ID, their median intensity values were considered. The parent genes to which the different protein groups were mapped to are equivalent to ‘canonical proteins’ in UniProt (https://www.uniprot.org/help/canonical_and_isoforms) and therefore the term protein abundance is used to describe the protein abundance of the canonical protein throughout the manuscript. A detailed flowchart of all post-processing steps is shown in S1 Fig.

### Protein abundance comparison across datasets

For comparison of protein abundances between the two groups of samples (secretome and solid tumor samples), the normalised iBAQ abundances were transformed into numerical bins. The abundances (in ppb) were ranked and grouped into 5 bins, wherein proteins with the lowest protein abundance values were in bin 1 and those with the highest abundance values were in bin 5. A Pearson correlation coefficient for all samples was calculated on pairwise complete observations of bin transformed iBAQ values in R. Samples were hierarchically clustered on columns and rows using Euclidean distances.

### Differentially expressed proteins and Gene Ontology (GO) analysis

Differentially expressed canonical proteins between (tumor and mucosa, adenoma and mucosa or adenoma and tumor) samples were determined by performing a t-test after ranked bin transformation. Benjamini-Hochberg procedure was used to control the FDR. To investigate the source-specific protein profile of each subgroup of the secreted samples group, we identified and further analysed proteins categorized as “enriched proteins”. “Enriched proteins” were those proteins uniquely quantified in one of the subgroups or proteins with median bin values higher than each median bin value of the rest of the subgroups. Gene ontology (GO) analysis was performed using g:Profiler [50] and Enrichr [51]. Only differentially expressed proteins or “Enriched proteins” were selected for the analysis.

### In silico validation and functional enrichment analysis

Images and quantifications derived from immunohistochemistry assays were obtained from the HPA [52]. When several antibodies were available for a given protein, the most representative was selected for further analysis. Semi-quantitative analysis (high, medium, low and not detected) performed and available in HPA was used in this study.

The protein interactome was obtained using the STRING database. To restrict the interactome to the CRC context, only proteins with a strong Pearson correlation (p<0.01) with the protein of interest in the solid tumor samples were selected. Next, proteins with at least one medium confidence interaction (STRING score>0.4) were selected for the final interactome. The interactome was plotted and a GO functional enrichment analysis was performed.

### Transcriptomics data selection and processing

RNA-seq data generated on the Illumina HiSeq platform for 592 colorectal tumor samples from TCGA COADREAD dataset [53] was downloaded from cBioPortal [54]. Data was mapped with the RSEM (RNA-Seq by Expectation-Maximization) algorithm and normalized using the [log_2_(RSEM+1)] method as previously described [55]. RNA-seq data of samples from CPTAC (90 patients) were included in the mentioned TCGA COADREAD dataset. However, both proteomics and RNA-seq data were acquired only for CPTAC samples.

For primary tumor and normal mucosa comparison, GSE41258 [56], a large dataset containing 390 samples from 276 CRC patients, was selected from Gene Expression Omnibus (GEO). This dataset was selected because contains data from 190 primary tumor and 54 healthy mucosa samples obtained from individuals, including a high level of metadata annotation (age, sex, stage, recurrence, location, etc). Data was derived from 299 U133A arrays and processed and normalized according to the original publication [56].

### Proteomics and transcriptomics data comparison

For the comparison between healthy mucosa and tumor samples, proteomics and transcriptomics data were obtained from the solid samples batch and from the GSE41258 dataset, respectively. In both cases, a fold-change (tumor *vs* mucosa) and an associated p-value were obtained. For the protein expression, the data was converted into ranked bins as previously described. The p-value was obtained by a t-test and subsequent Benjamini-Hochberg correction. The fold change was obtained as the ratio of the means of the tumor and mucosa subgroups. Regarding transcriptomics, the fold change was obtained as the ratio of the normalized expression of the primary tumor and healthy mucosa subgroups. The p-value was also obtained by a t-test and subsequent Benjamini-Hochberg correction. The correlation between both fold changes was examined using scatter and volcano plots. Only differentially expressed canonical proteins were analysed. For the comparison of hazard ratios (HR) obtained by proteomics and transcriptomics, proteomics data from the CPTAC dataset and RNA-seq data from the TCGA and CPTAC datasets were used. The correlation between HR was examined using scatter plots. Only significant proteins using the Cox regression analysis were plotted.

### Statistical analysis

The significance of protein expression differences between groups was obtained by using two-sample t tests for each protein or gene. ANOVA tests were performed in order to detect significant differences in the risk-score between three or more groups. Block design was used to correct the variability corresponding to the batch effects. Univariate Cox regression and Kaplan-Meier analysis were performed using the ‘survival’ and ‘survminer’ R packages (https://CRAN.R-project.org/package=survival). Intensity values were transformed to a z-score before survival analysis. For Kaplan-Meier analysis, patients were divided into two subgroups (high or low expression) by the optimal cut-off method, using Cutoff Finder [57]. The point at which the log-rank test split was most significant was identified as the optimal cut-off.

### Integration into Expression Atlas

In Expression Atlas, protein expression data coming from the individual reanalysis of each dataset is available. The calculated canonical protein abundances (mapped as genes), and summary files detailing the quality of post-processing of all datasets were integrated as proteomics baseline experiments (E-PROT identifiers are available in Table 1). It should be noted that Expression Atlas mainly provides a dataset centric view, so this is why the protein abundance data were integrated in that way. For the overall results described in the manuscript, the datasets were analysed in two batches (as explained above) to provide an improved control of the FDR at different levels (PSM and protein). However, it is important to highlight that the conclusions reached were the same with regards to the described identified biomarkers. They were also significantly associated to survival (in the CPTAC dataset) and overexpressed in blood coming from CRC patients (dataset PXD010458) when using the protein abundance results coming from the individual reanalyses of datasets (as integrated in Expression Atlas) instead of the combined reanalysis.

## Supporting information

S1 Table List of the proteins corresponding to the SEC6 signature. (DOCX)

S2 Table List of the proteins identified as new potential biomarkers. (DOCX)

S1 Fig Detailed flow chart of the meta-analysis study. (SDRF: Sample Data Relationship Format, IDF: Investigation Description Format).
(TIF)

S2 Fig Heatmap of binned protein abundances in colorectal tumor samples. Non-hierarchical clustering of the solid samples according to Pearson correlation. Dataset and sample subgroup are indicated.
(TIF)

S3 Fig Heat-map of binned protein abundances in colorectal cancer secreted samples. Non-hierarchical clustering of the secreted samples according to Pearson correlation. Dataset, conditions and sample subgroup are indicated.
(TIF)

S4 Fig Enriched proteins in secreted samples. (A) Heat-map representing the Pearson coefficient between fold changes of solid and secreted samples. (B) Enriched canonical proteins in each subgroup. Proteins are considered to be enriched when quantified only in one subgroup or expression is at least double than the rest of subgroups. (C) Gene ontology (Biological Process) analysis of the enriched proteins indicating more relevant categories. (D) EC vesicle category from Cellular Component GO analysis in the enriched proteins.
(TIF)

S5 Fig Extended analysis of prognosis association. (A) Kaplan–Meier analysis of high- and low-expression patients in stage II and III. P values were obtained by log-rank test. (B) Classification of patients in high or low risk according to the expression of 5 different biomarkers. (C) Kaplan–Meier analysis of high- and low risk patients in all the stages (left) and stages II and III (right). Patients are considered as high risk when at least 3 biomarkers are classifying them as high risk.
(TIFF)

S6 Fig Distribution of the selected biomarkers in transcriptomics. (A) Kaplan–Meier analysis of high- and low-expression patients using the complete TCGA COADREAD (n = 431). P values were obtained by log-rank test. (B) Forest plots of HRs associated to each gene (TCGA) or protein (CPTAC) in each dataset. P values were obtained by Cox regression analysis. (C) Log-rank analysis of the biomarkers according to eight different transcriptomics datasets.
(TIF)

S7 Fig Immunohistochemistry analysis according to Human Protein Atlas (HPA). (A) Representative PPIA, PRDX1, TXNDC5, MRC2 and CD14 protein expression by IHC in CRC cases according to HPA series of cases. (B) Percent of CRC cases expressing high, medium, low or null levels of protein according to HPA. Most representative antibodies were selected for the analysis.
(TIF)
