# Supplementary material for: Integrated meta-analysis of colorectal cancer public proteomic datasets for biomarker discovery and validation
Source: PLoS Comput Biol. 2024 Jan 22;20(1):e1011828. doi: 10.1371/journal.pcbi.1011828 (PMC10833860; doi:10.1371/journal.pcbi.1011828)
Supplement: S1 Table — (DOCX) [file pcbi.1011828.s001.docx]

**Table S1.** List of the proteins corresponding to the SEC6 signature

| Gene symbol | Protein name | UniprotKB | Mol. Weight (kDa) | Amino acids |
| --- | --- | --- | --- | --- |
| BMP1 | Bone morphogenetic protein 1 | P13497 | 111.25 | 968 |
| CD109 | CD109 antigen | Q6YHK3 | 161.69 | 1445 |
| IGFBP3 | Insulin-like growth factor-binding protein 3 | P17936 | 63.35 | 291 |
| LTBP1 | Latent-transforming growth factor beta-binding protein 1 | Q14766 | 186.79 | 1721 |
| NPC2 | NPC intracellular cholesterol transporter 2 | P61916 | 16.57 | 151 |
| PSAP | Prosaposin | P07602 | 58.11 | 524 |
