# Supplementary material for: Integrated meta-analysis of colorectal cancer public proteomic datasets for biomarker discovery and validation
Source: PLoS Comput Biol. 2024 Jan 22;20(1):e1011828. doi: 10.1371/journal.pcbi.1011828 (PMC10833860; doi:10.1371/journal.pcbi.1011828)
Supplement: S2 Table — (DOCX) [file pcbi.1011828.s002.docx]

**Table S2.** List of the proteins identified as new potential biomarkers.

| Gene symbol | Protein name | UniprotKB | Mol. Weight (kDa) | Amino acids |
| --- | --- | --- | --- | --- |
| CD14 | Monocyte differentiation antigen CD14 | P08571 | 40.08 | 375 |
| MRC2 | C-type mannose receptor 2 | Q9UBG0 | 166.67 | 1479 |
| PPIA | Peptidyl-prolyl cis-trans isomerase A | P62937 | 18.01 | 165 |
| PRDX1 | Peroxiredoxin-1 | Q06830 | 22.11 | 199 |
| TXNDC5 | Thioredoxin domain-containing protein 5 | Q8NBS9 | 48.62 | 432 |
