# Supplementary figures and images for: Integrated meta-analysis of colorectal cancer public proteomic datasets for biomarker discovery and validation
Source: PLoS Comput Biol. 2024 Jan 22;20(1):e1011828. doi: 10.1371/journal.pcbi.1011828 (PMC10833860; doi:10.1371/journal.pcbi.1011828)

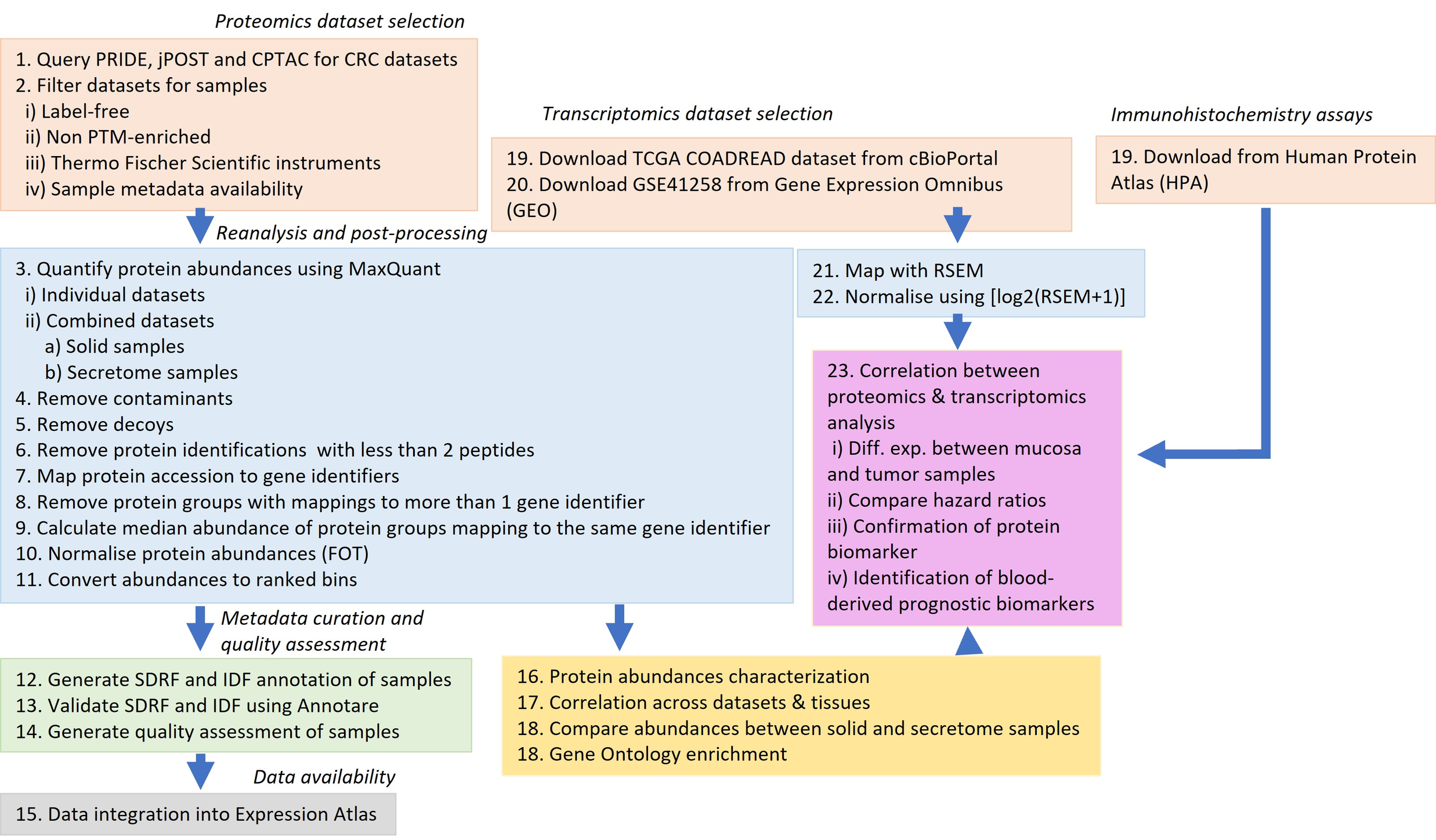

Supplement: S1 Fig — (SDRF: Sample Data Relationship Format, IDF: Investigation Description Format). (TIF) [file pcbi.1011828.s003.tif]

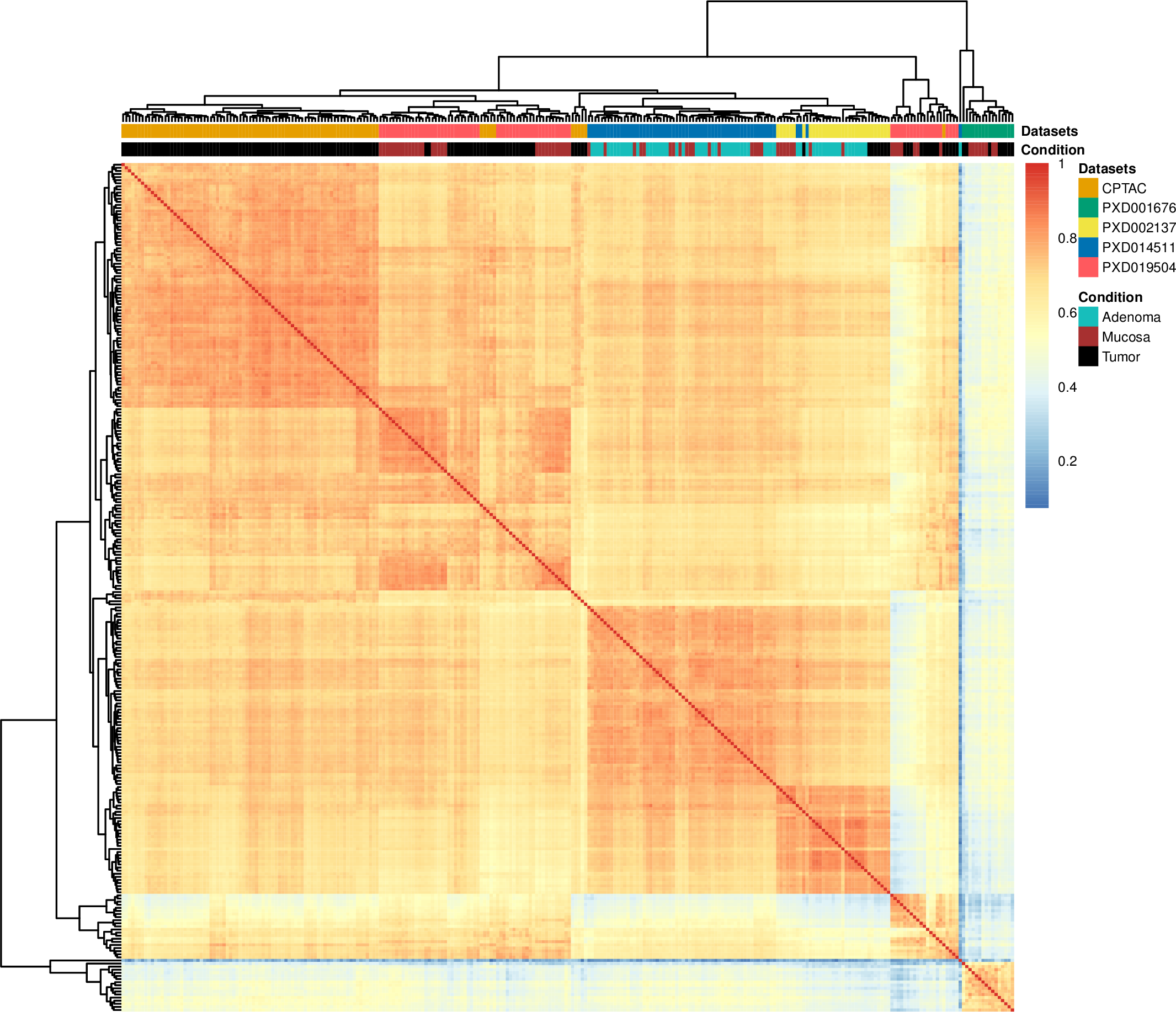

Supplement: S2 Fig — Non-hierarchical clustering of the solid samples according to Pearson correlation. Dataset and sample subgroup are indicated. (TIF) [file pcbi.1011828.s004.tif]

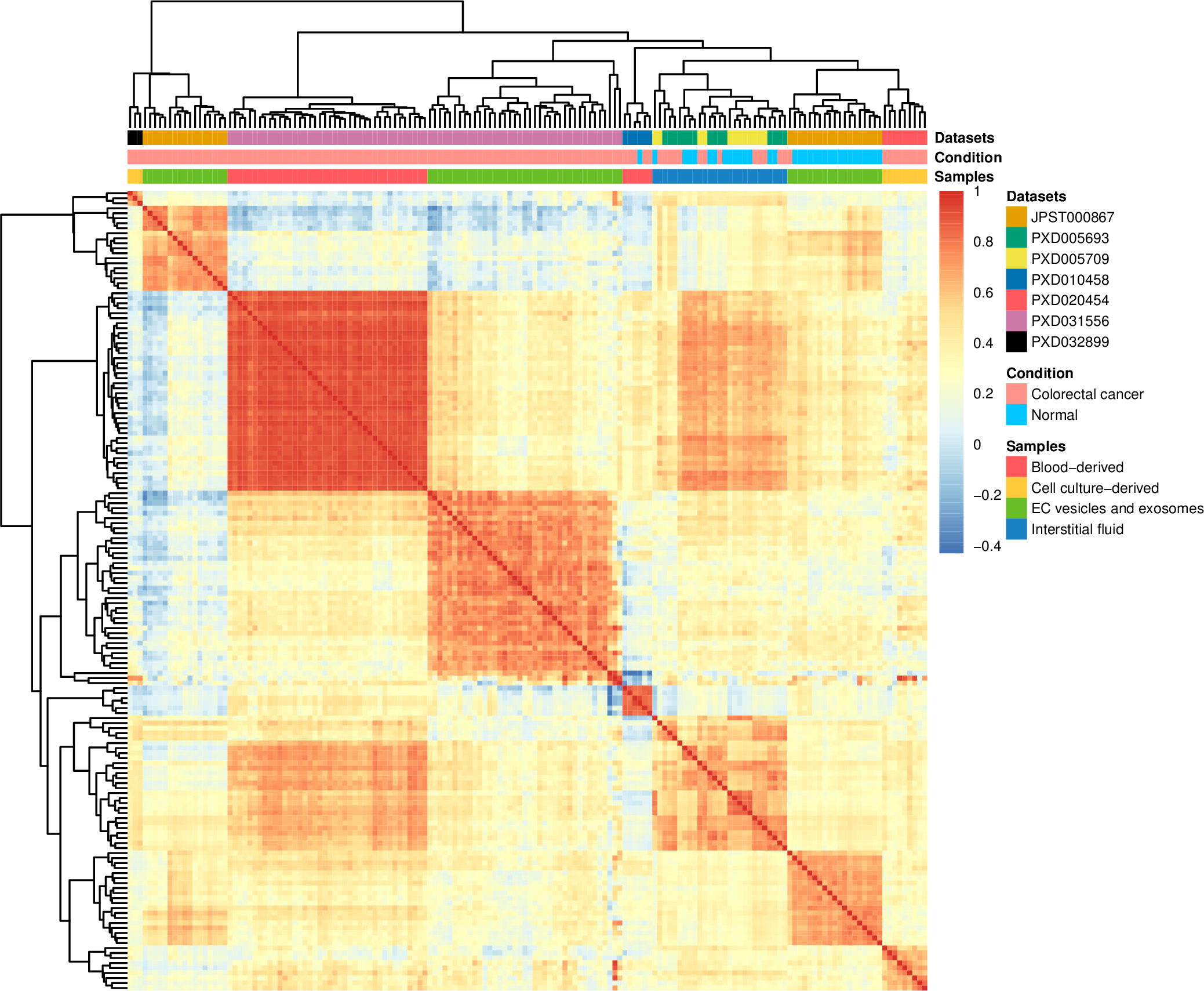

Supplement: S3 Fig — Non-hierarchical clustering of the secreted samples according to Pearson correlation. Dataset, conditions and sample subgroup are indicated. (TIF) [file pcbi.1011828.s005.tif]

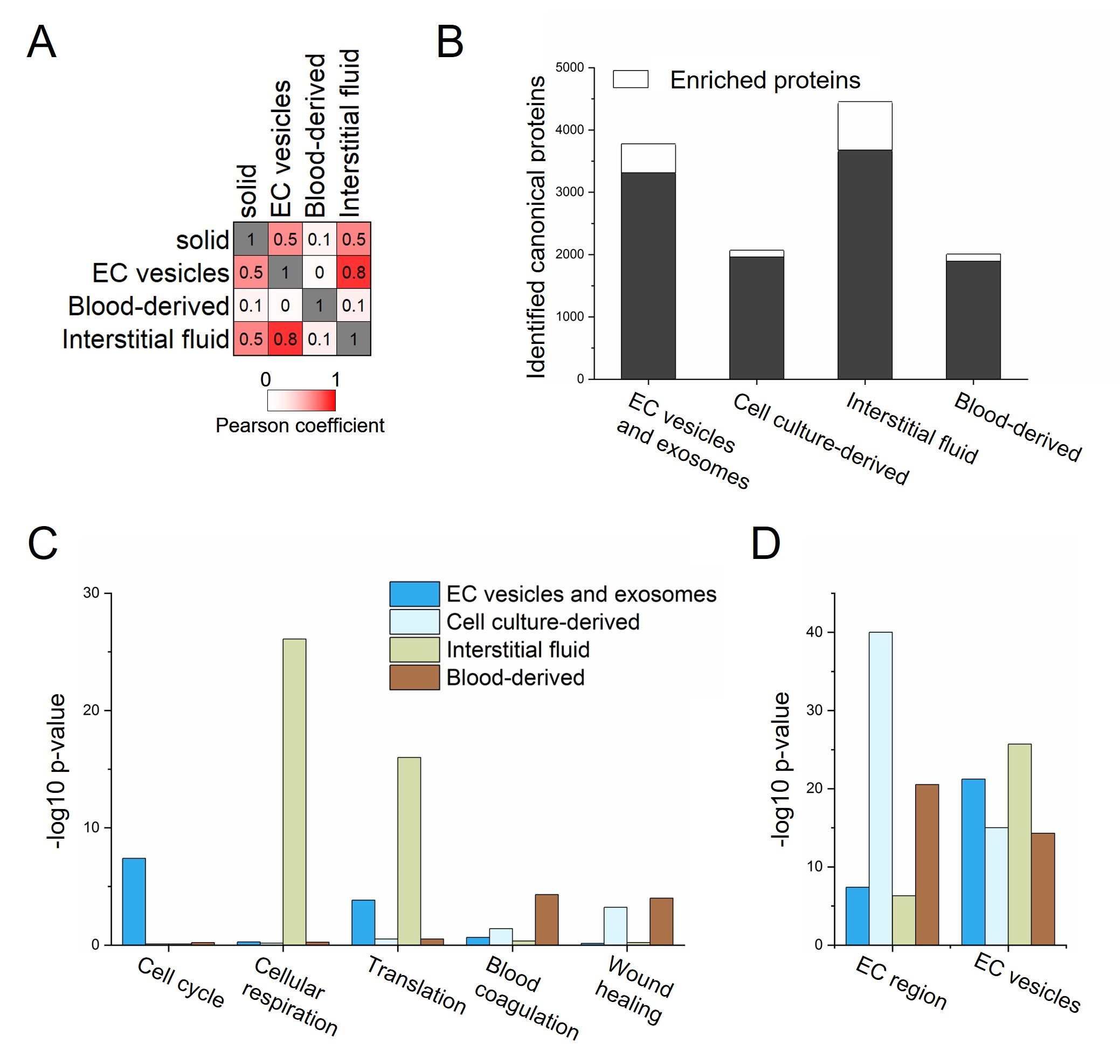

Supplement: S4 Fig — (A) Heat-map representing the Pearson coefficient between fold changes of solid and secreted samples. (B) Enriched canonical proteins in each subgroup. Proteins are considered to be enriched when quantified only in one subgroup or expression is at least double than the rest of subgroups. (C) Gene ontology (Biological Process) analysis of the enriched proteins indicating more relevant categories. (D) EC vesicle category from Cellular Component GO analysis in the enriched proteins. (TIF) [file pcbi.1011828.s006.tif]

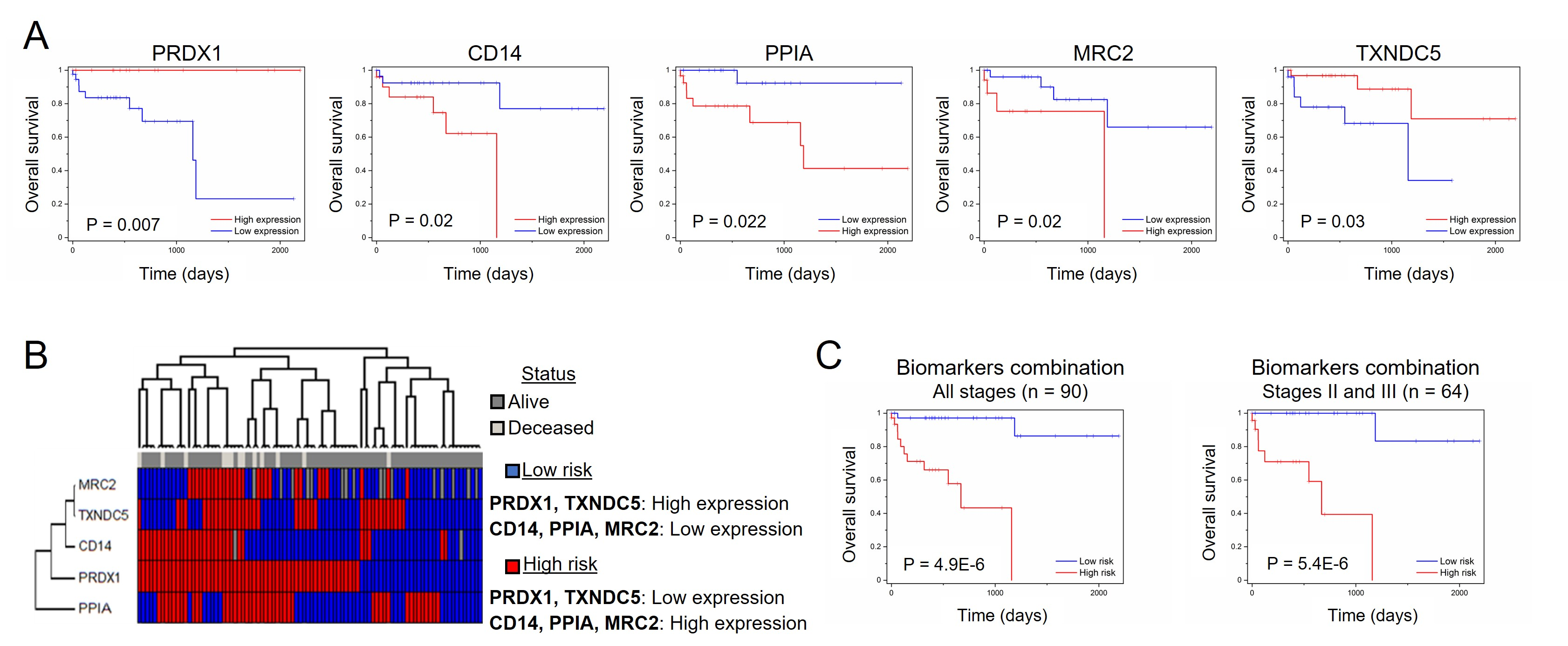

Supplement: S5 Fig — (A) Kaplan–Meier analysis of high- and low-expression patients in stage II and III. P values were obtained by log-rank test. (B) Classification of patients in high or low risk according to the expression of 5 different biomarkers. (C) Kaplan–Meier analysis of high- and low risk patients in all the stages (left) and stages II and III (right). Patients are considered as high risk when at least 3 biomarkers are classifying them as high risk. (TIFF) [file pcbi.1011828.s007.tiff]

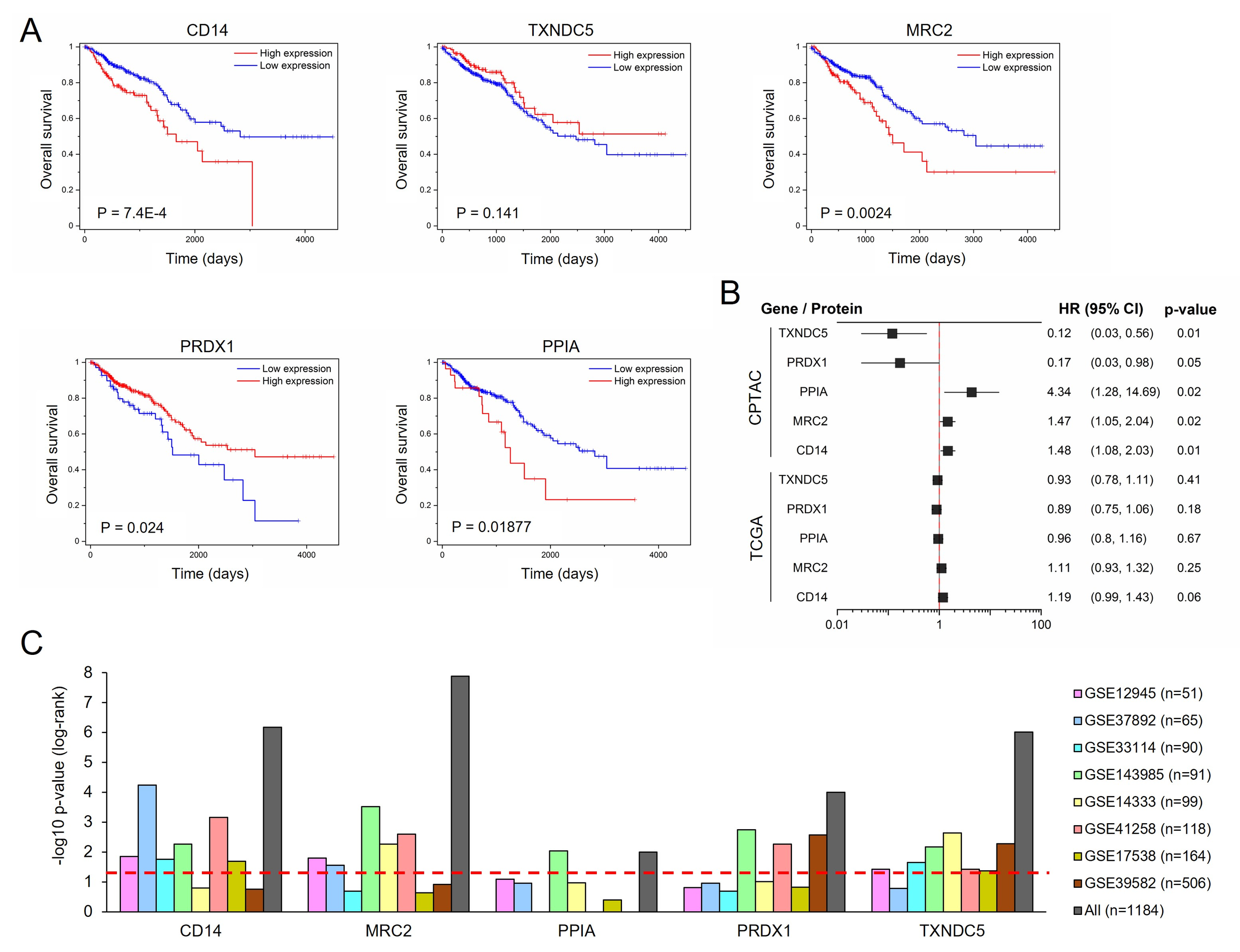

Supplement: S6 Fig — (A) Kaplan–Meier analysis of high- and low-expression patients using the complete TCGA COADREAD (n = 431). P values were obtained by log-rank test. (B) Forest plots of HRs associated to each gene (TCGA) or protein (CPTAC) in each dataset. P values were obtained by Cox regression analysis. (C) Log-rank analysis of the biomarkers according to eight different transcriptomics datasets. (TIF) [file pcbi.1011828.s008.tif]

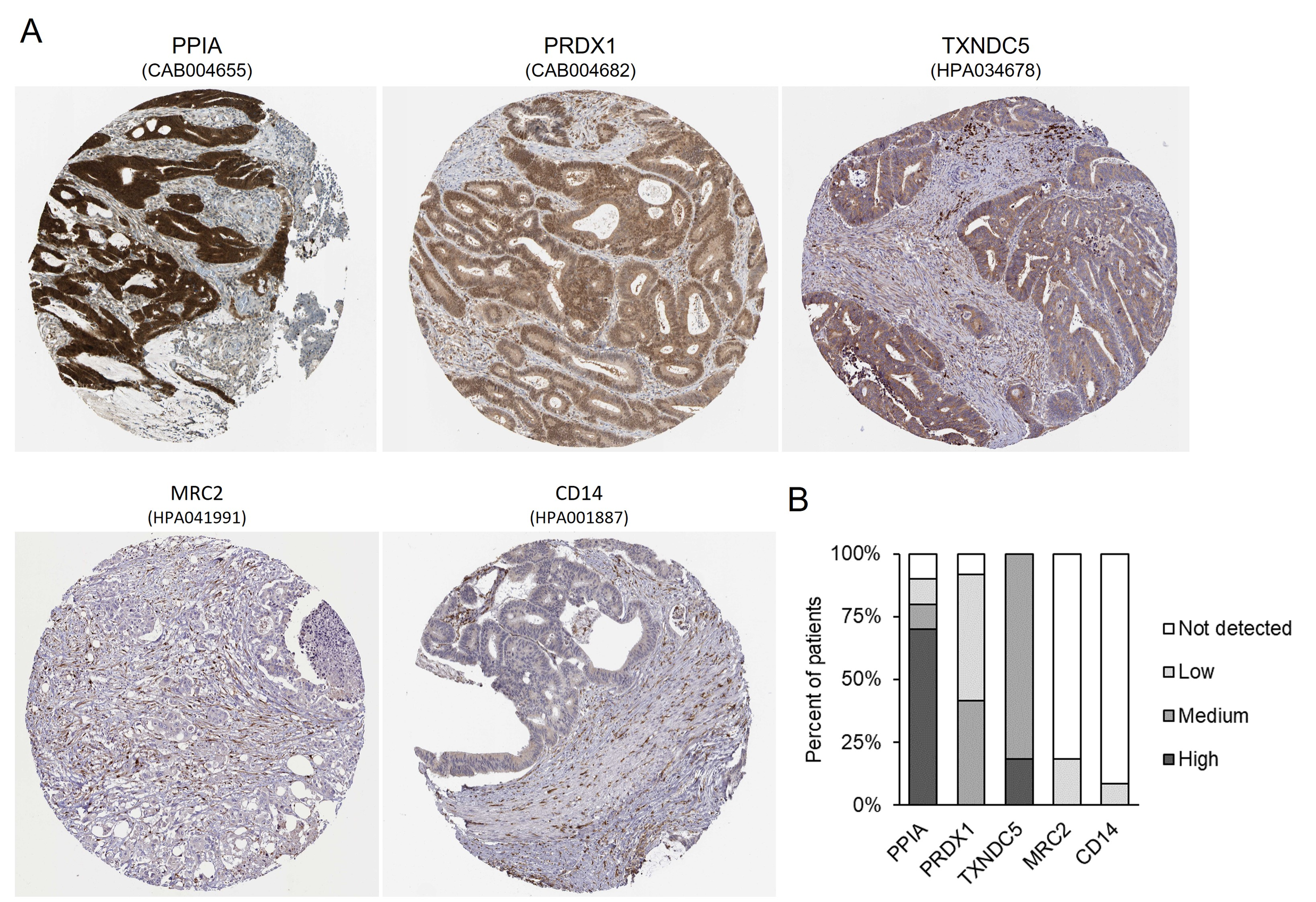

Supplement: S7 Fig — (A) Representative PPIA, PRDX1, TXNDC5, MRC2 and CD14 protein expression by IHC in CRC cases according to HPA series of cases. (B) Percent of CRC cases expressing high, medium, low or null levels of protein according to HPA. Most representative antibodies were selected for the analysis. (TIF) [file pcbi.1011828.s009.tif]
